# Global research landscape, knowledge structure, and emerging trends in adverse childhood experiences and personality disorders: a bibliometric analysis

**DOI:** 10.3389/fpsyt.2026.1842315

**Published:** 2026-06-24

**Authors:** Liuyin Jin, Hua Xia, Lulu Sun

**Affiliations:** The Second People’s Hospital of Lishui, Lishui, China

**Keywords:** adverse childhood experiences, bibliometrics, borderline personality disorder, citespace, co-citation analysis, personality disorders, research hotspots, VOSviewer

## Abstract

**Background:**

The relationship between adverse childhood experiences (ACEs) and personality disorders (PDs) has attracted sustained attention in psychiatry, psychology, and public health. Existing studies have mainly examined epidemiological associations, specific PDs diagnoses, or mechanisms, whereas bibliometric evidence mapping the field’s knowledge structure and thematic evolution remains limited. This study aimed to characterize trends, contributors, collaboration networks, core themes, and frontiers in ACEs–PDs research.

**Methods:**

English-language publications on ACEs and PDs were retrieved from Web of Science Core Collection, Scopus, and PubMed from inception to December 31, 2025. After year screening, document-type filtering, and deduplication, 5,084 records were included. Bibliometric analyses were performed using R, VOSviewer, and CiteSpace. The merged dataset was used to examine annual trends, countries/regions, institutions, authors, journals, and keyword co-occurrence, while WoSCC records were used for co-citation analysis, keyword clustering, and burst detection.

**Results:**

ACEs–PDs research showed sustained growth, with a marked increase after 2000. The United States occupied a central position in publication output, citation impact, and international collaboration, while the United Kingdom, Germany, Canada, the Netherlands, and Australia also showed strong influence. Harvard University, the University of London, and Ruprecht Karls University Heidelberg were leading institutions; Zanarini M, Fonagy P, Schmahl C, Paris J, and Kleindienst N were key contributors. Influential journals mainly covered psychiatry, personality disorders, child maltreatment, trauma, and developmental psychopathology. Keyword analyses identified childhood adversity, personality disorder, borderline personality disorder, depression, childhood sexual abuse, and post-traumatic stress disorder as core themes. VOSviewer and CiteSpace analyses indicated that hotspots have expanded from childhood abuse, PDs diagnosis, and psychiatric comorbidity to emotion dysregulation, non-suicidal self-injury, social support, functional connectivity, early intervention, and mechanism validation. Highly cited publications revealed a knowledge base centered on childhood abuse/trauma, borderline personality disorder, psychiatric comorbidity, emotion regulation, and neurobiological mechanisms.

**Conclusion:**

This study maps development and knowledge structure of ACEs–PDs research. Findings suggest a shift from exposure–outcome association studies toward comorbidity, intermediate phenotypes, neurobiological mechanisms, and clinical translation. Future research should strengthen longitudinal and cross-cultural designs, consider ACE type, timing, duration, and severity, and integrate neuroimaging, inflammatory, epigenetic, and clinical-course phenotypes.

## 1 Introduction

Personality refers to a relatively stable pattern of psychological and behavioral characteristics, the formation of which is shaped by both genetic predispositions and early environmental experiences. Previous studies have shown that individual differences in personality have a certain genetic basis, with approximately 40% of the variance attributable to genetic factors, while the remaining variance is mainly related to environmental and other non-genetic factors (1). Personality traits may emerge early in life in the form of temperament and show a certain degree of stability during development, thereby providing an early foundation for the subsequent formation of personality structure (2). However, environmental exposures during development are equally important. Early trauma or chronic stress during sensitive developmental periods may affect brain maturation and stress-response systems, altering children’s emotional reactivity, impulse control, and social information processing, thereby increasing the likelihood of hypervigilance, heightened sensitivity, or aggressive behavioral tendencies (3). These characteristics may, in turn, influence the caregiving environment, for example by increasing conflicts with caregivers and punitive interactions, thus forming a developmental cascade of “individual characteristics–environmental responses–further adversity exposure” and producing cumulative effects at the neurobiological level (4, 5). Therefore, from the perspectives of developmental psychopathology and the biopsychosocial model, personality disorders (PDs) can be understood as the outcome of long-term interactions among biological vulnerability, early environmental stress, and subsequent social adaptation processes. Under specific genetic backgrounds and early experiential conditions, individuals may develop relatively stable cognitive, emotional, and interpersonal coping patterns to adapt to their family and social environments. When these patterns become increasingly rigid and lead to persistent, pervasive, and clinically significant functional impairment, they may manifest as personality pathology or personality disorders (6–8).

Among various early environmental exposures, adverse childhood experiences (ACEs) generally refer to a range of unfavorable experiences encountered during early development, including emotional neglect, physical or sexual abuse, family dysfunction, poverty, inadequate caregiving, and domestic violence. A large body of evidence indicates that ACEs, as important environmental risk factors, may produce long-term cumulative effects and significantly increase the risk of PDs in adulthood. Some studies have reported that any type of childhood adversity may increase the risk of personality disorders, with the risk rising by up to approximately 3.8-fold (9, 10). In recent years, discussions regarding the conceptual boundaries of ACEs have continued to expand. Some studies have suggested that bullying, community violence, exposure to domestic violence, non-medical trauma related to chronic childhood illness, and unpredictable early-life environments may also constitute broader forms of childhood adversity (11, 12). Further epidemiological and clinical evidence consistently supports a stable association between childhood abuse/neglect and PDs, with a certain dose–response pattern: the greater the number and severity of adversity types, the higher the risk of PDs in adulthood (7, 13, 14). Different types of childhood adversity may be differentially associated with distinct personality disorder phenotypes. Previous studies have shown that multiple forms of childhood adversity are common in the general population, are more prevalent among individuals with PDs, and are particularly strongly associated with schizotypal, antisocial, borderline, and narcissistic personality disorders (15, 16). After controlling for other types of childhood adversity, mood disorders, anxiety disorders, substance use disorders, other personality disorder types, and sociodemographic variables, childhood abuse and neglect remained significantly associated with Cluster A and Cluster B personality disorders, with odds ratios ranging from 1.22 to 1.63. Mood disorders, anxiety disorders, and substance use disorders were also significantly associated with various personality disorder types (OR = 1.26–2.38), suggesting that the relationship between ACEs and PDs is often intertwined with a complex background of psychiatric comorbidity (17). Notably, increasing evidence suggests that “time does not heal all wounds”: even in adulthood or old age, childhood adversity remains associated with a higher likelihood of mood disorders, anxiety disorders, and PDs (18). In addition, a meta-analysis focusing on Cluster C personality disorders also suggested a significant association between adverse childhood events and anxiety-, avoidance-, and dependency-related personality pathology (19). Umbrella reviews have further indicated that childhood abuse, neglect, and other early adverse environmental factors are important risk factors for multiple PDs (20).

From the perspective of developmental psychopathology, ACEs may shape personality pathology through multiple interrelated mechanisms. At the psychosocial level, early adversity may promote the formation of insecure attachment, weaken emotion regulation capacity, reinforce negative self-schemas, and impair interpersonal trust, thereby laying a vulnerable foundation for later interpersonal sensitivity, emotional instability, and impulsivity (21, 22). At the neurobiological level, prolonged early-life stress may affect limbic–prefrontal regulatory circuits and reward-related networks, thereby altering key processing phenotypes such as threat processing, reward processing, emotion regulation, and executive control. Specifically, studies on threat processing have shown that maltreated individuals may exhibit either enhanced or reduced neural responses in regions such as the amygdala. The former is commonly interpreted as hypervigilance to threat cues, whereas the latter may reflect adaptive or defensive processing patterns such as avoidance or emotional numbing (23, 24). Findings on reward processing have more consistently indicated that maltreated individuals may show reduced responses during reward anticipation and reward receipt, particularly reflected in decreased striatal activity; this pattern is often associated with anhedonia and depressive symptoms. Studies on emotion regulation have further reported increased activation of the anterior cingulate cortex (ACC) during active emotion regulation tasks, which may indicate that individuals require greater cognitive resources to suppress emotional responses (25). Meanwhile, executive control studies have reported increased dorsal ACC activity during error monitoring and inhibitory control tasks, suggesting the coexistence of increased monitoring/inhibitory load and reduced processing efficiency (26, 27). Beyond these neural circuits and processing phenotypes, endocrine–immune–molecular pathways may also serve as key mediators linking ACEs and personality disorders, including dysregulation of the hypothalamic–pituitary–adrenal (HPA) axis, chronic low-grade inflammation, and epigenetic alterations (28–32).

Bibliometrics is a quantitative research method that uses visual and statistical analyses of publication trends, author collaboration networks, keyword co-occurrence patterns, and highly cited literature to reveal the developmental trajectory, knowledge structure, and emerging frontiers of a given research field from a macro-level perspective (33, 34). In recent years, bibliometric methods have been widely applied in psychiatry and psychology. However, systematic bibliometric analyses focusing on the interdisciplinary topic of “ACEs and PDs” remain limited. Unlike previous narrative reviews, systematic reviews, and meta-analyses, the present study aims to comprehensively map the developmental trajectory, major contributors, collaboration patterns, core themes, and thematic evolution of this field from a macro-level knowledge-mapping perspective.

Therefore, based on the Web of Science Core Collection (WoSCC), Scopus, and PubMed databases, this study systematically retrieved and integrated relevant publications published up to December 31, 2025, and conducted multi-level bibliometric analyses using R, VOSviewer, and CiteSpace. Considering the differences among databases in literature coverage and citation data structure, the merged dataset from the three databases was used for descriptive and thematic analyses, including annual publication trends, countries/regions, institutions, authors, journals, and keywords. Meanwhile, because WoSCC provides more standardized and complete reference fields, the WoSCC dataset was further used for citation network analyses, including co-citation analysis, clustering, and burst detection.

Through this hierarchical analytical strategy, this study aims to more comprehensively reveal the knowledge structure, research hotspots, and future directions of ACEs and PDs research, thereby providing a reference for subsequent mechanistic studies, clinical translation, and interdisciplinary collaboration.

## 2 Methods

### 2.1 Study design and analytical framework

This study employed bibliometric methods to systematically analyze publication trends, knowledge structure, collaboration networks, research hotspots, and thematic evolution in studies related to ACEs and PDs. Considering the differences among databases in literature coverage, completeness of citation fields, and reference formats, a hierarchical analytical strategy was adopted.

Specifically, records retrieved from the WoSCC, Scopus, and PubMed were converted, merged, cleaned, and deduplicated, and were then used for descriptive bibliometric analyses, including annual publication trends, countries/regions, institutions, authors, journals, keywords, and collaboration networks. Analyses that depend on complete reference and citation relationship data, such as co-citation analysis, citation network analysis, and burst detection, were performed based on the WoSCC dataset.

The rationale for adopting this hierarchical strategy was that merging records from three databases could broaden literature coverage and reduce omissions caused by relying on a single database, whereas WoSCC provides relatively standardized cited reference fields and is therefore more suitable for co-citation networks, clustering analysis, and burst detection in CiteSpace. PubMed does not provide complete citation relationship data, and although Scopus contains citation information, its reference format, indexing scope, and citation-counting system differ from those of WoSCC. Directly merging Scopus with WoSCC for citation network analysis may lead to duplicate citations, inconsistent reference identification, and biased network structures (35–38). Therefore, in this study, Scopus and PubMed were mainly used to expand the data coverage for descriptive analyses, while WoSCC was used to maintain the consistency and reproducibility of citation network analyses (36, 39).

### 2.2 Data sources and search strategy

This study searched three databases: WoSCC, Scopus, and PubMed. Database retrieval and data export were completed on April 26, 2026. It should be noted that this date refers to the date of database retrieval and data export, and does not indicate that publications from 2026 were included. To avoid time-truncation bias caused by incomplete annual data and delays in database indexing, this study included only publications published up to December 31, 2025. Records published in 2026, online-first records with an official publication year of 2026, and records with missing publication years that could not be confirmed were excluded during data cleaning.

The search strategy consisted of two thematic modules: terms related to adverse childhood experiences and terms related to personality disorders. The two modules were connected using “AND,” while terms within each module were connected using “OR,” to ensure that retrieved records simultaneously involved both adverse childhood experiences and personality disorder-related topics.

Terms related to adverse childhood experiences included “adverse childhood experiences,” “childhood adversity,” “childhood trauma,” “childhood maltreatment,” “childhood abuse,” “childhood neglect,” “early life stress,” and related expressions. Terms related to personality disorders included “personality disorder,” “personality pathology,” “personality dysfunction,” and expressions related to specific personality disorder types (12, 40). The Topic field was used in WoSCC, the Title, Abstract, and Keywords fields were used in Scopus, and MeSH terms together with Title/Abstract fields were used in PubMed. The complete search strategies are provided in the **Supplementary Materials**.

### 2.3 Inclusion and exclusion criteria

This study is a bibliometric study rather than a systematic review or meta-analysis. Thematic relevance was mainly controlled using the predefined search strategy, which simultaneously included terms related to adverse childhood experiences and terms related to personality disorders connected by “AND.” This study did not conduct full-text eligibility assessment in the manner of a systematic review, nor did it further exclude studies based on full-text content.

The inclusion criteria were as follows:

Records retrieved from the WoSCC, Scopus, or PubMed databases;

Records matching both adverse childhood experience-related terms and personality disorder-related terms in the search strategy;

Document type classified as Article or Review;

Publication date no later than December 31, 2025;

English-language publications.

The exclusion criteria were as follows:

Records published after 2025 or records with missing publication years that could not be confirmed;

Records with document types other than Article or Review;

Duplicate records;

Records with severely incomplete database export information that could not be used for subsequent bibliometric analysis.

### 2.4 Data conversion, merging, and deduplication

After data export, records from WoSCC, Scopus, and PubMed were imported into R software for format conversion, field harmonization, and merging. Since the field names and formats exported from different databases varied, fields including authors, titles, journals, publication years, DOIs, abstracts, keywords, countries/regions, and institutions were standardized before merging.

The literature screening and deduplication process mainly included publication year screening, document type screening, and duplicate removal. First, records were screened according to publication year, retaining only records published no later than December 31, 2025 with clear year information. Subsequently, records were screened by document type, and only Articles and Reviews were retained.

Deduplication was performed using a hierarchical strategy. First, records with DOIs were standardized and deduplicated based on DOI. For records without DOIs, title, first author, and publication year were combined for assisted deduplication. Finally, an additional cross-database title–year assisted deduplication step was performed to reduce residual duplicates caused by missing DOIs, differences in title capitalization, punctuation variations, or inconsistencies in database record formats. The complete literature retrieval, screening, and deduplication process is presented in a PRISMA-like flow diagram.

### 2.5 Descriptive bibliometric analysis

Based on the final merged dataset from WoSCC, Scopus, and PubMed, descriptive bibliometric analyses were conducted. The analyses included annual publication trends, major countries/regions, institutions, authors, journals, cited publications, keyword frequencies, and collaboration relationships.

Annual publication trends were used to illustrate the developmental stages and growth patterns of the field. Analyses of countries/regions, institutions, and authors were used to identify the main contributors and their collaboration relationships. Journal analysis was used to evaluate major publication sources and academic dissemination channels. Keyword analysis was used to identify research hotspots and thematic structures.

During data cleaning, fields such as countries/regions, institutions, authors, and keywords were standardized to reduce biases caused by differences in capitalization, abbreviations, synonyms, and database labeling. For example, country/region names were harmonized for visualization and statistical analysis: the United States was uniformly labeled as “USA,” China was uniformly labeled as “China,” and other country names were presented according to standard English names.

### 2.6 VOSviewer visualization analysis

VOSviewer was used to construct keyword co-occurrence networks, country/region collaboration networks, institutional collaboration networks, and related visual maps (41). To ensure the interpretability of network structures and avoid overly crowded figures, corresponding thresholds were set according to different analytical objects.

For keyword co-occurrence analysis, the analysis type was set as Co-occurrence, the unit of analysis was set as All keywords, and the counting method was Full counting. No additional thesaurus file was imported into VOSviewer. The minimum occurrence threshold for keywords was set to 50. In the network map, nodes represent keywords, node size indicates keyword frequency, links between nodes represent keyword co-occurrence relationships, and thicker links indicate stronger co-occurrence strength. In the overlay visualization, node color represents the average year of keyword occurrence, which was used to show the temporal evolution of research hotspots.

For institutional collaboration network analysis, the analysis type was set as Co-authorship, the unit of analysis was Organizations, and the counting method was Full counting. To avoid excessive influence of papers with extremely large numbers of collaborating institutions on the network structure, the option “Ignore documents co-authored by a large number of organizations” was selected, and the maximum number of organizations allowed per document was set to 25. The institutional inclusion threshold was set as a minimum of 15 publications and a minimum of 0 citations. In the network map, nodes represent institutions, node size indicates institutional publication output, links represent collaboration relationships between institutions, and link thickness reflects collaboration strength. Except for the threshold settings described above, the network layout, clustering, and visualization parameters in VOSviewer were kept at the software default settings.

### 2.7 CiteSpace citation network and burst analysis

CiteSpace was used to conduct citation network analysis, co-citation analysis, keyword clustering, and burst detection based on the WoSCC dataset (42). Since co-citation analysis and burst detection rely on complete and consistently formatted references and citation relationships, this part of the analysis used only WoSCC data to ensure the internal consistency and reproducibility of the citation network structure.

According to the CiteSpace settings used in this study, the time span was set from January 2015 to December 2025, with a time slice of 1 year. Text sources included Title, Abstract, Author Keywords, and Keywords Plus. Node types were set according to the specific analytical purpose: Keyword was selected for keyword analysis, and Reference was selected for co-citation analysis. Link strength was measured using Cosine, and the network scope was set as Within Slices. Node selection was based on the g-index, with the scale factor k set to 25. Burst detection was used to identify keywords or references with rapidly increasing frequency or citation strength during specific periods, reflecting stage-specific changes in research hotspots in this field.

This study did not include publications from 2026. Therefore, the time range in CiteSpace-related analyses, figures, tables, and textual descriptions was consistently set as 2015–2025 (43, 44).

### 2.8 Quality control and reproducibility

To improve research transparency and reproducibility, the following quality control measures were adopted. First, the complete search strategies for each database are provided separately in the **Supplementary Materials** to facilitate replication of the retrieval process. Second, the literature retrieval, screening, and deduplication process is presented in a PRISMA-like flow diagram, with the number of records and reasons for exclusion indicated at each step. Third, during data cleaning, DOIs, titles, authors, publication years, countries/regions, institutions, and keywords were standardized to reduce biases caused by duplicate records and inconsistent naming. Fourth, descriptive analyses and keyword co-occurrence analyses were performed based on the merged three-database dataset, whereas citation network, co-citation, and burst analyses were performed based on the WoSCC dataset; this hierarchical analytical strategy and its rationale were explicitly described in the Methods section. Finally, all major software parameters, including the analysis types, counting methods, and threshold settings in VOSviewer, as well as the time span, time slicing, node types, link strength, and selection criteria in CiteSpace, were reported in the Methods section to improve the interpretability and reproducibility of the findings.

## 3 Results

### 3.1 Literature retrieval, screening, and deduplication

A total of 8,756 original records were retrieved from the WoSCC, Scopus, and PubMed databases. Among them, 2,470 records were obtained from WoSCC, 3,671 from Scopus, and 2,615 from PubMed. All records were restricted to English-language publications, with publication dates up to December 31, 2025, and only Articles and Reviews were retained.

After data merging, records were first screened according to publication year. A total of 50 records published after 2025 or with missing publication years were excluded, leaving 8,706 records. Subsequently, document type screening was performed, and 151 records that were not classified as Articles or Reviews were excluded. The remaining 8,555 records entered the deduplication process.

**Figure 1:**
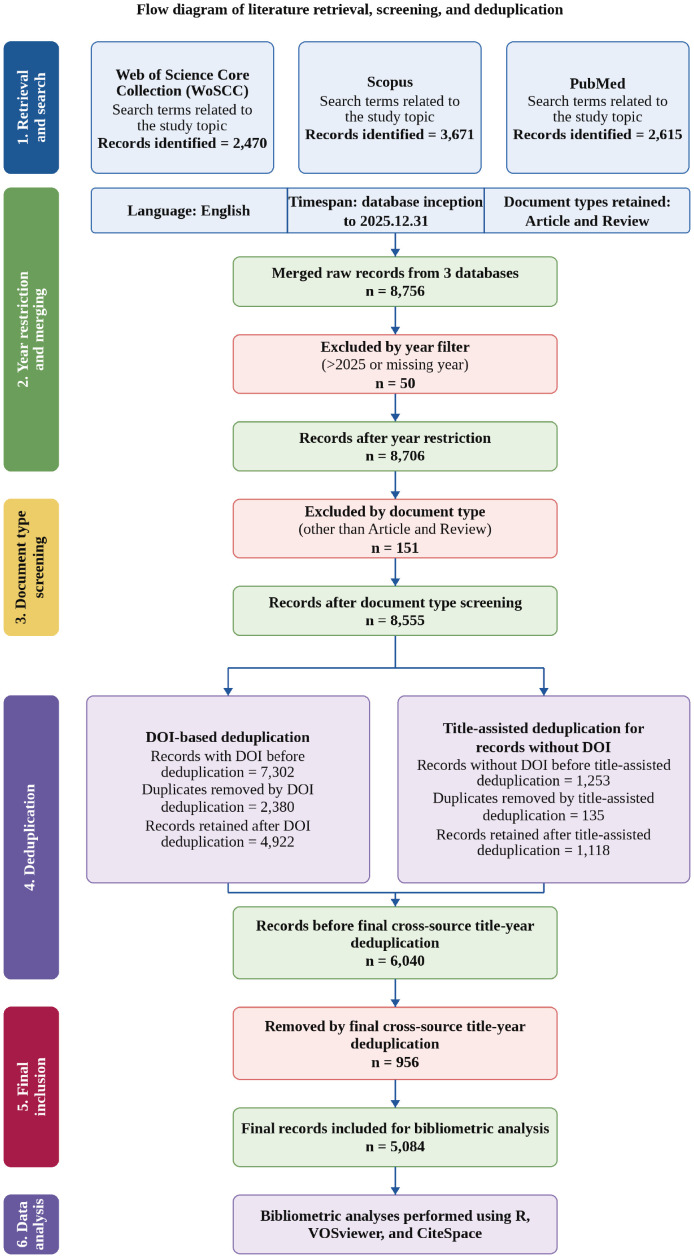
Flowchart of literature retrieval and selection.

A hierarchical deduplication strategy was adopted. First, DOI-based deduplication was conducted for records with available DOIs. Among 7,302 records with DOIs, 2,380 duplicate records were removed, and 4,922 records were retained. For records without DOIs, title-assisted deduplication was performed. Among 1,253 records without DOIs, 135 duplicate records were removed, and 1,118 records were retained. The records retained after DOI-based deduplication and title-assisted deduplication were then merged, yielding 6,040 records. A further cross-database title–year assisted deduplication step was conducted, removing 956 additional duplicate records. Ultimately, 5,084 publications were included for subsequent bibliometric analysis. The literature retrieval, screening, and deduplication process is shown in **Figure 1**.

### 3.2 Annual publication trends in ACEs and PDs research

**Figure 2:**
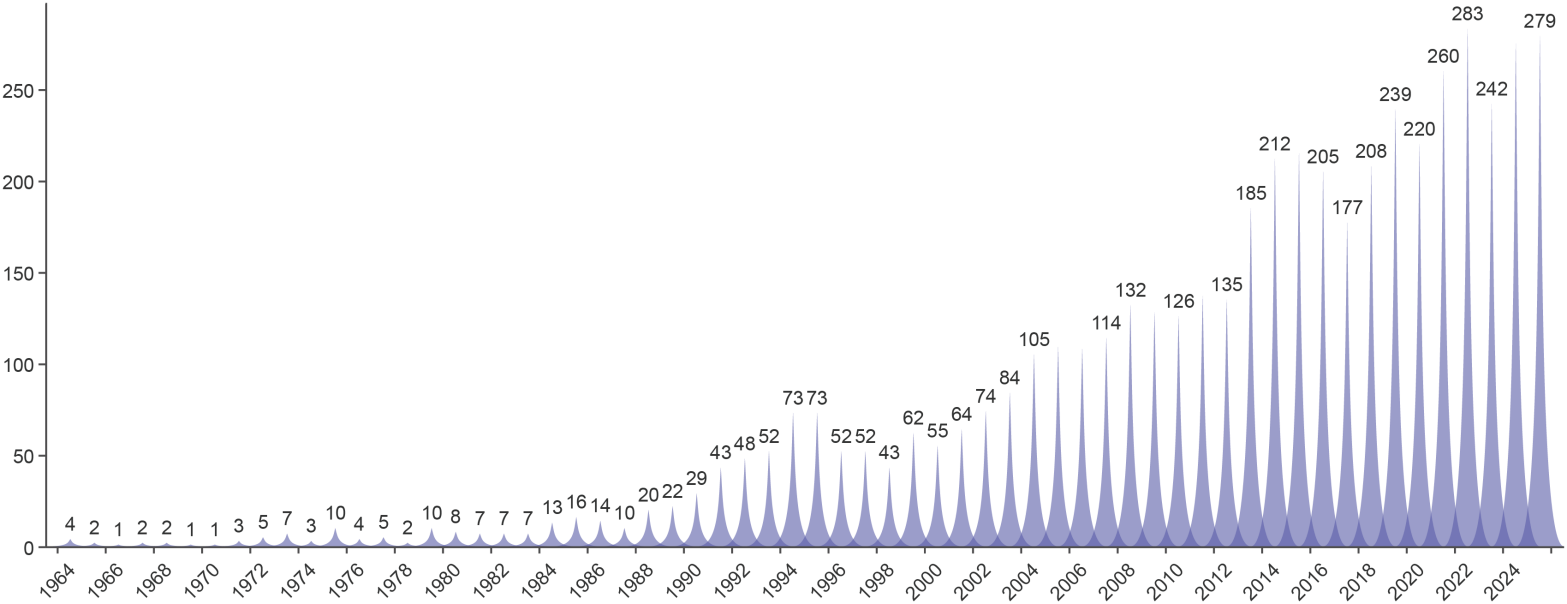
Annual publication trends in the field of childhood adversity and personality disorders research (1964–2026).

As shown in **Figure 2**, the annual number of publications on childhood adversity and personality disorders showed an overall upward trend. The earliest publication in the dataset appeared in 1964. During the early stage, particularly from the 1960s to the 1970s, the number of publications remained low, with most years contributing fewer than 10 articles, indicating that this topic had not yet formed a stable research field.

From the 1980s to the 1990s, research output gradually increased, rising from only a small number of annual publications to several dozen publications per year. This suggests that the relationship among childhood adversity, traumatic experiences, and personality disorders began to receive increasing academic attention. After 2000, publication output continued to grow, and this growth became more pronounced after 2010. Since 2013, the annual number of publications has remained at a relatively high level, with a marked increase observed from the late 2010s to the 2020s. In recent years, annual output has consistently exceeded 200 publications, reaching a peak of 283 publications in 2023 and remaining high in 2025 with 279 publications.

Overall, the field has evolved from an early exploratory stage to a period of gradual accumulation and, more recently, rapid growth. The substantial increase in publications in recent years indicates that the association between childhood adversity and personality disorders has become an increasingly important research topic in psychiatry, psychological trauma, and public health.

### 3.3 Distribution and collaboration patterns of ACEs and PDs publications across countries/regions

**Table 1** presents the top 10 countries/regions ranked by publication output in the field of adverse childhood experiences and personality disorders. The USA ranked first, with 1,566 publications, demonstrating its dominant position in this field. Among these publications, the USA had 1,061 single-country publications (SCPs) and 505 multiple-country publications (MCPs), with an MCP proportion of 32.2%. The United Kingdom ranked second, with 794 publications, followed by Germany with 554, Canada with 501, the Netherlands with 379, Australia with 349, Italy with 249, Spain with 182, Switzerland with 174, and China with 156.

**Table 1:** National contributions and international collaboration patterns in research on childhood adversity and personality disorder.

Rank	Country/Region	Publications	SCP	MCP	MCP (%)
1	United States	1566	1061	505	32.2
2	United Kingdom	794	342	452	56.9
3	Germany	554	251	303	54.7
4	Canada	501	285	216	43.1
5	Netherlands	379	169	210	55.4
6	Australia	349	179	170	48.7
7	Italy	249	122	127	51.0
8	Spain	182	68	114	62.6
9	Switzerland	174	32	142	81.6
10	China	156	80	76	48.7

SCP, single-country publications; MCP, multiple-country publications. Obvious duplicate country/region labels were harmonized before ranking; non-country strings and address/email fragments were excluded.

Regarding international collaboration, Switzerland had the highest MCPs proportion, reaching 81.6%, indicating that its research in this field was highly dependent on international collaboration. Spain also showed a high MCPs proportion of 62.6%. The United Kingdom, the Netherlands, Germany, and Italy had MCPs proportions of 56.9%, 55.4%, 54.7%, and 51.0%, respectively, all showing active cross-national collaboration. In contrast, although the USA had a much higher publication output than other countries, its MCPs proportion was only 32.2%, suggesting that a large proportion of its research output originated from domestic collaboration networks. China ranked tenth, with 156 publications and an MCPs proportion of 48.7%, indicating that China has established a certain research foundation in this field and has a moderate level of participation in international collaboration.

Overall, research on adverse childhood experiences and personality disorders has been mainly driven by North American and European countries. The USA has a clear advantage in publication volume, while European countries such as Switzerland, Spain, the United Kingdom, the Netherlands, and Germany show stronger international collaboration characteristics. These findings indicate that this field includes both highly productive research forces centered on the USA and an increasingly developed transnational collaboration network in which European countries serve as important nodes.

### 3.4 Top 10 source journals by publication output

**Table 2** presents the top 10 source journals ranked by publication output. Overall, studies on adverse childhood experiences and personality disorders were mainly published in journals related to psychiatry, personality disorders, trauma, child abuse, affective disorders, and developmental psychopathology. This suggests that the field has clear interdisciplinary characteristics, involving psychiatry, clinical psychology, trauma research, violence and abuse studies, and developmental psychopathology.

**Table 2:** Top 10 source journals.

Source	NP	PY start
Psychiatry Research	123	1989
Journal of Personality Disorders	112	1991
Journal of Interpersonal Violence	95	1999
Journal of Nervous and Mental Disease	92	1979
Child Abuse and Neglect	87	1983
Journal of Affective Disorders	87	1988
Comprehensive Psychiatry	80	1977
American Journal of Psychiatry	65	1981
Development and Psychopathology	64	1998
Journal of Psychiatric Research	60	1992

TC, total citations; NP, number of publications; PY start, publication year of first record.

In terms of publication output, Psychiatry Research published the largest number of relevant articles, with 123 publications, making it the most important publication source in this field. It was followed by the Journal of Personality Disorders, with 112 publications, suggesting that specialist journals on personality disorders occupy an important position in ACEs–PDs research. The Journal of Interpersonal Violence ranked third, with 95 publications, reflecting the close relationship among interpersonal violence, trauma exposure, childhood adversity, and personality disorder-related topics. The Journal of Nervous and Mental Disease, Child Abuse & Neglect, and the Journal of Affective Disorders also published a relatively large number of relevant studies, with 92, 87, and 87 publications, respectively.

In terms of journal type, the presence of Child Abuse & Neglect indicates that child abuse and neglect constitute an important research foundation in this field. The Journal of Affective Disorders reflects that ACEs–PDs research is often intertwined with affective disorders such as depression and anxiety, as well as psychiatric comorbidity. Development and Psychopathology highlights the important role of the developmental psychopathology perspective in explaining how early adversity contributes to personality pathology.

Regarding the starting publication year, Comprehensive Psychiatry was the earliest journal to publish relevant studies, with its first publication in this field appearing in 1977. It was followed by the Journal of Nervous and Mental Disease in 1979 and the American Journal of Psychiatry in 1981. This suggests that general psychiatry journals began to pay attention to the relationship between adverse childhood experiences and personality disorders relatively early. Overall, Psychiatry Research, the Journal of Personality Disorders, and the Journal of Interpersonal Violence are the most active publication sources in this field, while Child Abuse & Neglect, Development and Psychopathology, and the Journal of Affective Disorders reflect the close connections between this topic and child trauma, developmental psychopathology, and psychiatric comorbidity research.

### 3.5 International collaboration network analysis

**Figure 3:**
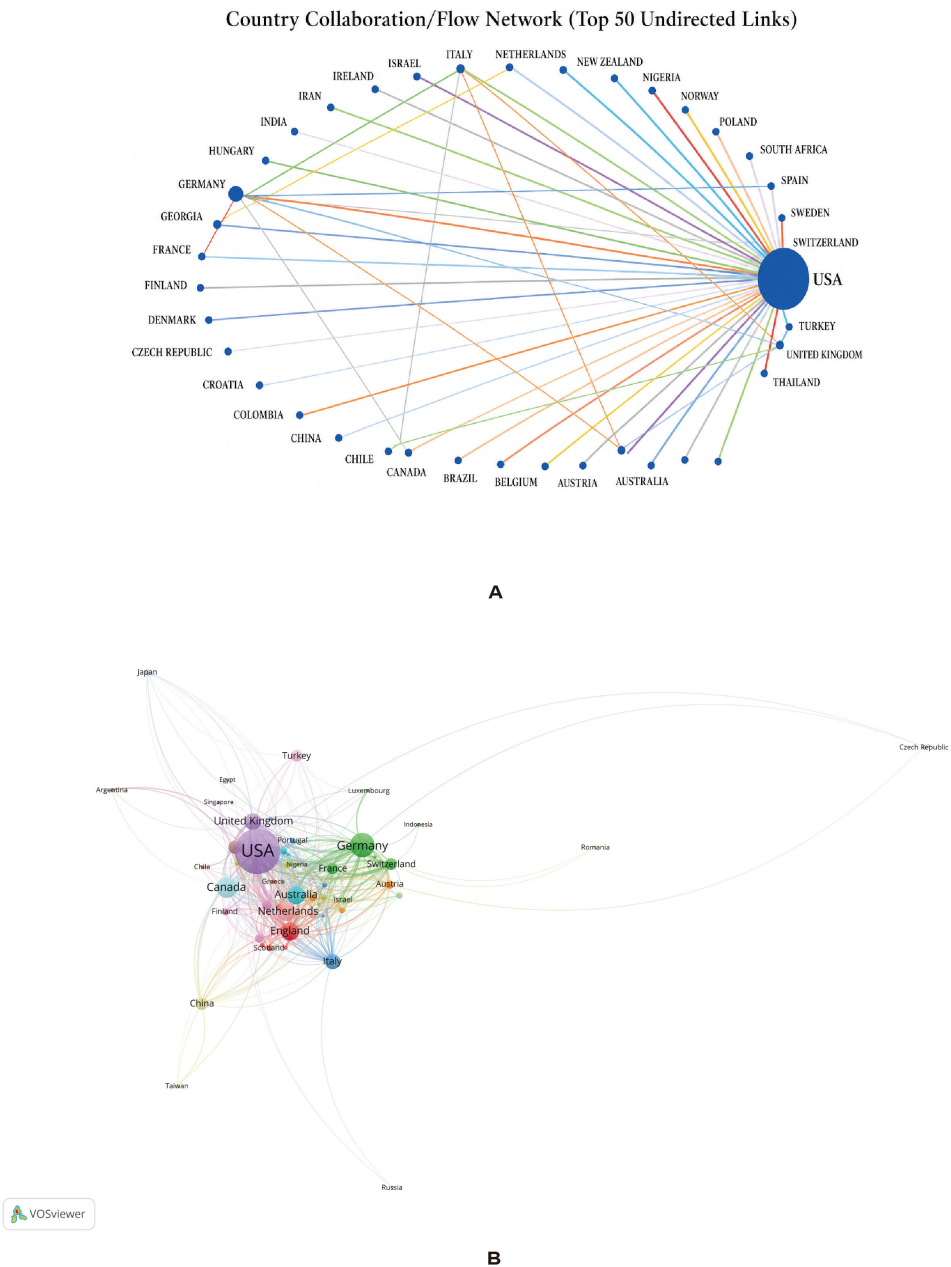
Country collaboration network and collaboration flow analysis based on three databases. **(A)** Country collaboration/flow network (Top 50 undirected links): nodes represent countries and links represent international collaborations; thicker links indicate stronger collaboration. The plot displays the top 50 undirected collaboration links with the highest strength. **(B)** VOSviewer visualization of the country collaboration network: node size indicates publication output (or total link strength, depending on the setting), links represent collaborations, and inter-node distance reflects relatedness; colors denote collaboration clusters.

As shown in **Figure 3**, a relatively extensive international collaboration network has formed in the field of adverse childhood experiences and personality disorders. **Figure 3A** presents the top 50 country/region collaboration links, while **Figure 3B** further shows the country/region collaboration network constructed using VOSviewer. Overall, the collaboration network displayed a clear core–periphery structure. The USA occupied the central position in the network, had the largest node, and established strong collaborative links with multiple countries/regions. This indicates that the USA not only dominates in terms of publication output but also serves as a central hub in the international collaboration network of this field.

**Figure 3A** shows that the USA had frequent collaboration links with the United Kingdom, Germany, Canada, Australia, the Netherlands, Switzerland, Italy, Spain, Sweden, and other countries, forming the major transnational collaboration pathways in this field. Germany, the United Kingdom, Canada, and the Netherlands also occupied relatively important positions in the network and maintained collaborative relationships with multiple countries/regions, suggesting close cooperation among European and North American countries. In contrast, although some Asian, South American, and African countries have entered the collaboration network, their nodes were smaller and their collaborative links were relatively limited, indicating that their participation in international collaboration in this field could be further strengthened.

The VOSviewer network in **Figure 3B** further shows that the USA, the United Kingdom, Germany, Canada, the Netherlands, Australia, Italy, and Switzerland formed a relatively close collaboration core. Different colors represent different collaboration clusters, indicating that international collaboration in this field was not randomly distributed but instead formed several collaboration groups based on geographic proximity, academic traditions, or long-term cooperative relationships. The dense connections among European countries suggest strong regional collaboration within Europe on this research topic. China was located in a relatively peripheral position in the network but still had collaborative links with the USA and some European countries, indicating that China has established a certain foundation for international collaboration in this field, although its overall collaboration strength remains relatively weaker than that of major research countries in Europe and North America.

Overall, the country/region collaboration network in this field is centered on the USA, with the United Kingdom, Germany, Canada, the Netherlands, Australia, and Switzerland jointly forming the main collaboration backbone. Research collaboration is mainly concentrated among countries in North America and Europe, while participation from other regions remains relatively limited. Future studies should further strengthen collaboration between core research countries and regions such as Asia, South America, and Africa to promote greater diversity and global representativeness of the evidence base in this field.

### 3.6 Publication output distribution of highly productive institutions and authors based on the integrated three-database datasets

**Figure 4:**
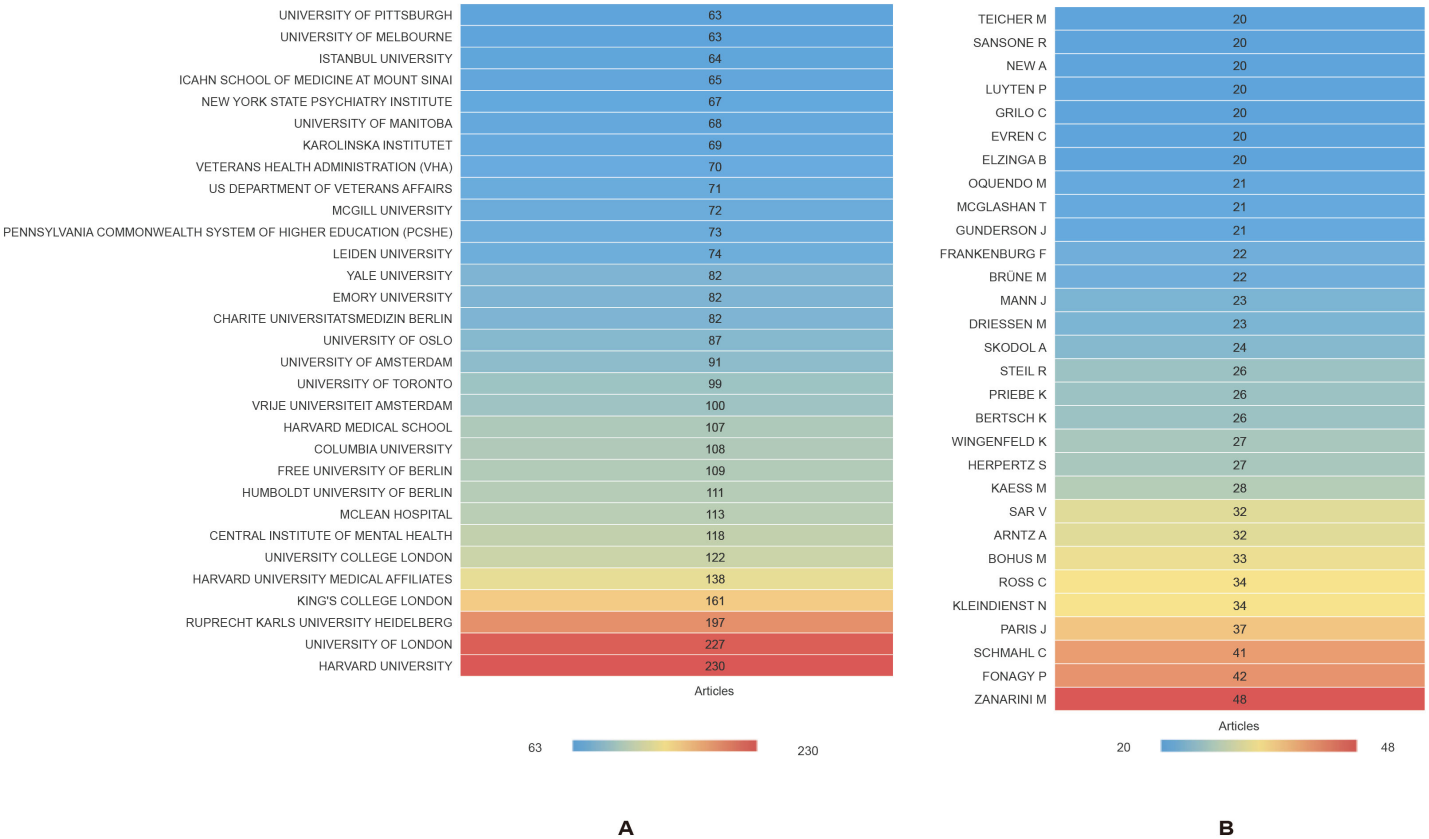
Publication output of the most productive institutions and authors based on integrated data from three databases. **(A)** Heatmap of article counts for the top institutions. **(B)** Heatmap of article counts for the top authors.

As shown in **Figure 4**, this study further analyzed highly productive institutions and authors in the field of adverse childhood experiences and personality disorders. **Figure 4A** presents the top 30 institutions ranked by publication output. Harvard University had the highest number of publications, with 230 articles, followed by the University of London with 227 articles and Ruprecht Karls University Heidelberg with 197 articles. Other leading institutions included King’s College London with 161 publications, Harvard University Medical Affiliates with 139, University College London with 122, Central Institute of Mental Health with 116, and McLean Hospital with 113. These institutions are mostly universities or medical centers with strong research capacity in psychiatry, psychology, medicine, and public health, suggesting that this field has a clear medicine–psychology interdisciplinary research profile.

**Figure 4B** shows the top 30 authors ranked by publication output. Zanarini M published the largest number of articles, with 48 publications, demonstrating a prominent contribution to this field. This was followed by Fonagy P with 42 publications, Schmahl C with 41, Paris J with 37, and Kleindienst N and Ross C with 34 publications each. Overall, highly productive institutions in this field were mainly concentrated in the USA and Europe, especially Harvard University, the University of London, Heidelberg University, and King’s College London. Highly productive authors were mainly active in research areas related to personality disorders, trauma-related psychopathology, borderline personality disorder, and the impact of childhood trauma. These findings suggest that several stable core research teams and academic centers have formed in the field of adverse childhood experiences and personality disorders.

### 3.7 Keyword distribution, co-occurrence network, and thematic evolution

**Figure 5:**
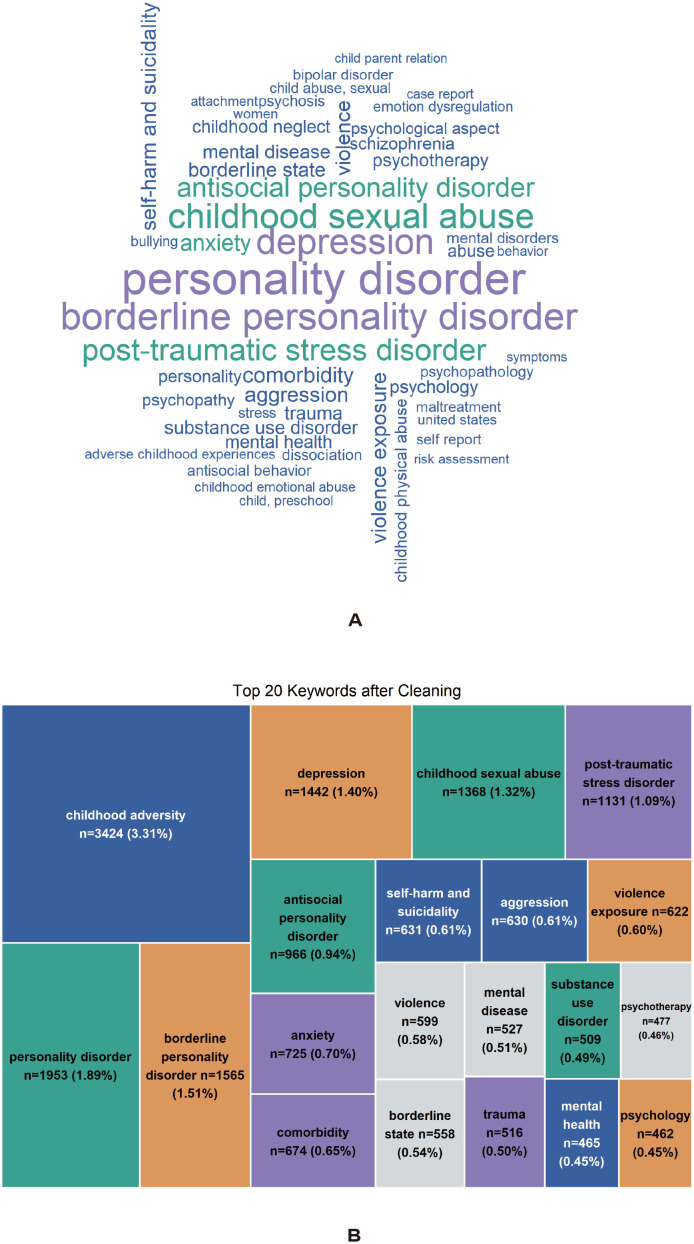
Keyword distribution and frequency. **(A)** Word cloud of keywords, where font size represents frequency of occurrence. **(B)** Treemap visualization of keyword frequencies, where rectangle size reflects the relative contribution of each keyword. Keywords were standardized prior to analysis. Visualization reflects thematic prominence rather than semantic relationships.

Based on the merged bibliographic dataset from WoSCC, Scopus, and PubMed, this study first analyzed the frequency distribution and co-occurrence network of cleaned keywords. **Figure 5** shows that high-frequency keywords in this field were mainly concentrated on adverse childhood experiences, personality disorders, and related psychiatric and psychological outcomes. In **Figure 5A**, terms such as “childhood sexual abuse,” “personality disorder,” “borderline personality disorder,” “depression,” and “post-traumatic stress disorder” were particularly prominent. **Figure 5B** further quantifies keyword frequency, with “childhood adversity” showing the highest frequency (n = 3,424, 3.31%), followed by “personality disorder” (n = 1,953, 1.89%), “borderline personality disorder” (n = 1,565, 1.51%), “depression” (n = 1,442, 1.40%), “childhood sexual abuse” (n = 1,368, 1.32%), and “post-traumatic stress disorder” (n = 1,131, 1.09%). In addition, keywords such as “antisocial personality disorder,” “anxiety,” “comorbidity,” “self-harm and suicidality,” “aggression,” “violence exposure,” “substance use disorder,” “psychotherapy,” and “trauma” also appeared frequently, indicating that this field not only focuses on the core association between adverse childhood experiences and personality disorders but also broadly involves clinically relevant issues such as depression, anxiety, post-traumatic stress, self-harm and suicidality, aggressive behavior, substance use, and psychiatric comorbidity.

**Figure 6:**
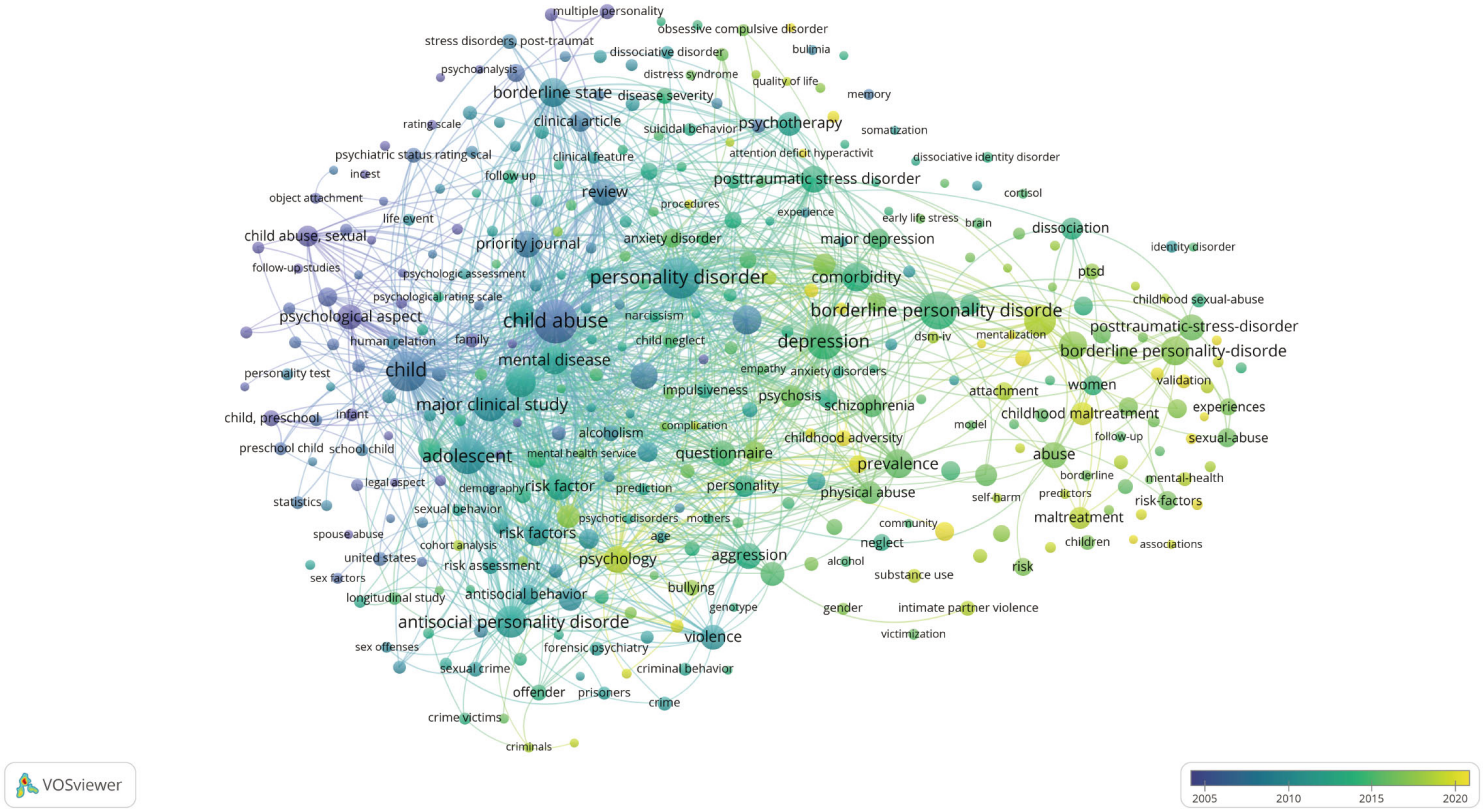
Overlay visualization of the keyword co-occurrence network generated by VOSviewer.

Further VOSviewer keyword co-occurrence analysis showed that studies on adverse childhood experiences and personality disorders formed a relatively dense thematic network (**Figure 6**). The central area of the network was mainly composed of keywords such as “personality disorder,” “borderline personality disorder,” “childhood sexual abuse,” “child abuse,” “depression,” “post-traumatic stress disorder,” “anxiety,” and “antisocial personality disorder,” indicating that these topics have long been core concerns in this field. In the overlay visualization, darker-colored keywords were mostly associated with earlier research topics, such as “child abuse,” “childhood trauma,” “dissociation,” “psychopathology,” and “dissociative identity disorder.” In contrast, lighter-colored keywords more often represented research directions that have been active in recent years, such as “adolescent,” “eating disorder,” “emotion dysregulation,” “self-harm,” “social support,” and “early intervention.” These results suggest that research hotspots in this field have gradually shifted from earlier descriptive studies of the relationships among childhood trauma, personality disorders, and related psychopathology toward directions with greater clinical translational relevance, such as emotion regulation, self-harm risk, adolescent populations, social support, and early intervention.

**Figure 7:**
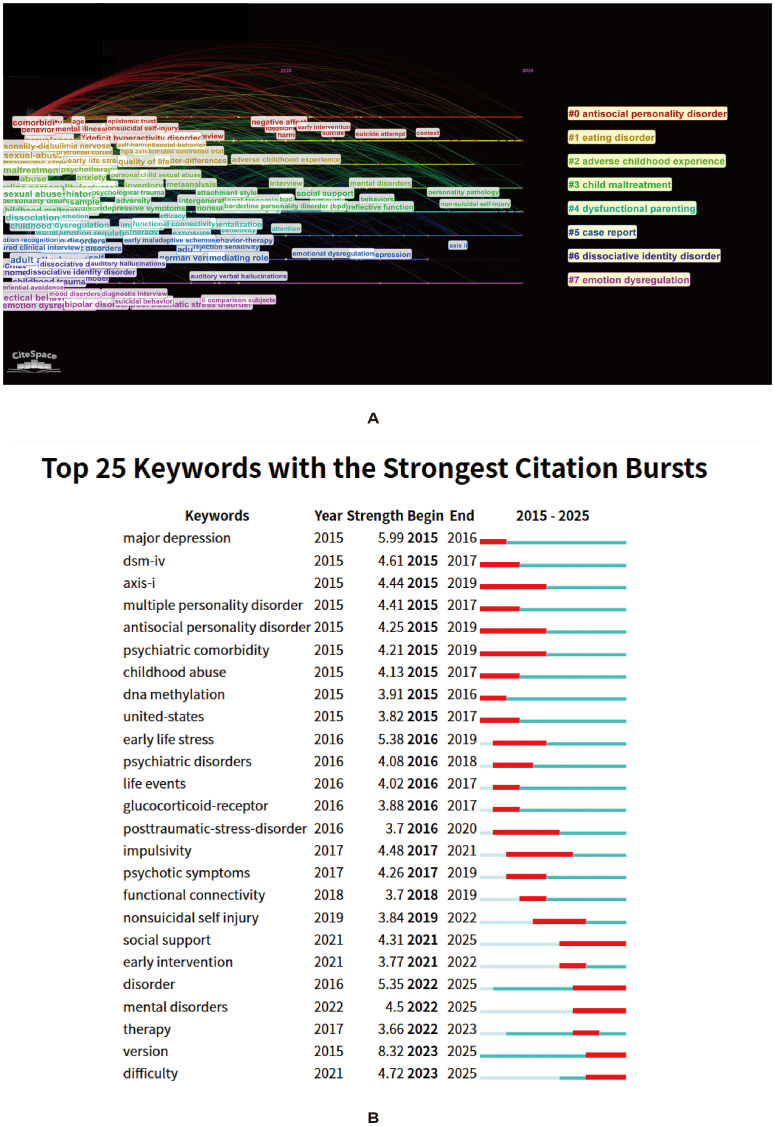
Keyword timeline clusters and burst terms based on the Web of Science Core Collection (WoSCC). **(A)** CiteSpace timeline visualization of keyword clusters. Each horizontal line represents a cluster (#0–#7), and nodes on the line indicate keywords appearing in the corresponding time period; links denote co-occurrence relationships among keywords. Cluster labels are shown on the right **(B)** Top 25 keywords with the strongest citation bursts (2015–2025). “Strength” indicates burst intensity, and the red segments on the timeline bars mark the burst periods (begin–end years), reflecting time intervals when a keyword received sharply increased attention.

To further verify the stability of keyword thematic evolution, this study used CiteSpace to perform keyword timeline clustering and burst term detection based on the WoSCC dataset, which may be regarded as a supplementary and sensitivity validation of the keyword analysis results from the merged three-database dataset. **Figure 7A** shows that keyword timeline clustering formed several major themes, including “antisocial personality disorder,” “eating disorder,” “adverse childhood experience,” “child maltreatment,” “dysfunctional parenting,” “case report,” “dissociative identity disorder,” and “emotion dysregulation.” These clusters were generally consistent with the high-frequency keywords and co-occurrence themes identified in the merged three-database analysis, indicating good stability of the core research themes in this field.

**Figure 7B** shows that keywords with strong burst intensity during 2015–2025 included “major depression,” “DSM-IV,” “axis-i,” “multiple personality disorder,” “antisocial personality disorder,” “psychiatric comorbidity,” and “childhood abuse,” reflecting that earlier research paid more attention to diagnostic classification, personality disorder types, psychiatric comorbidity, and childhood abuse-related issues. Subsequently, keywords such as “early life stress,” “posttraumatic stress disorder,” “impulsivity,” “psychotic symptoms,” “functional connectivity,” and “nonsuicidal self injury” emerged successively, indicating that research gradually expanded toward early-life stress, neuropsychological mechanisms, impulsivity, psychotic symptoms, and non-suicidal self-injury. Keywords that have remained in a burst state in recent years include “social support,” “mental disorders,” “disorder,” “version,” and “difficulty,” suggesting that current research increasingly focuses on social support, psychiatric comorbidity, updates to diagnostic tools, and the complexity of clinical identification and intervention. Overall, the keyword frequency and co-occurrence network results from the merged three-database dataset were generally consistent with the CiteSpace thematic evolution analysis based on WoSCC data, supporting the judgment of this study regarding the hotspot structure and evolutionary trends in this field.

### 3.8 Analysis of the top 20 most cited publications and top 50 most cited authors in ACEs and PDs research

**Figure 8:**
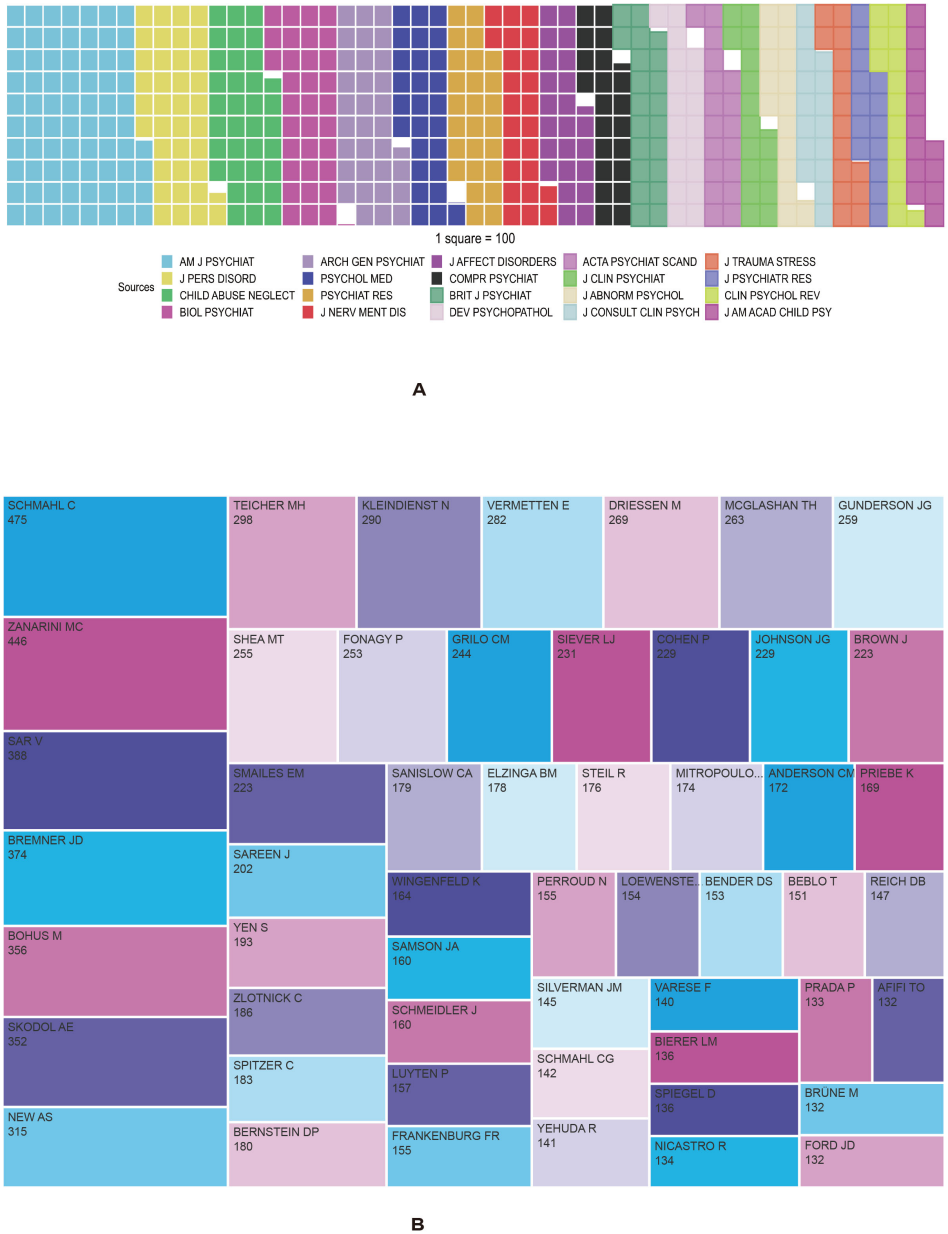
Top cited sources (Top 20 journals) and top cited authors (Top 50) in childhood adversity–personality disorder research. **(A)** Waffle plot of the top 20 cited sources (journals). One square represents 100 citations (1 square = 100), and different colors correspond to different journals **(B)** Treemap of the top 50 cited authors. Rectangle size and the embedded values indicate the number of citations; larger rectangles represent higher citation counts.

**Figure 8** presents the main cited sources and highly cited authors within the knowledge base of this field. **Figure 8A** shows that highly cited sources were mainly concentrated in journals related to psychiatry, personality disorders, child abuse and trauma, clinical psychology, and developmental psychopathology. Journals such as the American Journal of Psychiatry, Journal of Personality Disorders, Child Abuse & Neglect, Biological Psychiatry, Archives of General Psychiatry, Psychological Medicine, Psychiatry Research, and the Journal of Nervous and Mental Disease accounted for a large proportion, suggesting that the theoretical and empirical foundations of this field mainly originate from the intersection of psychiatry, trauma research, and personality disorder research.

**Figure 8B** further shows the distribution of highly cited authors. Authors with high citation frequencies included Schmahl C with 475 citations, Zanarini MC with 446, Sar V with 388, Bremner JD with 374, Bohus M with 356, Skodol AE with 352, and New AS with 315. In addition, Teicher MH, Kleindienst N, Vermetten E, Driessen M, McGlashan TH, and Gunderson JG also showed high influence. The research of these authors mainly focuses on borderline personality disorder, childhood trauma, post-traumatic stress disorder, dissociative symptoms, emotion dysregulation, and related psychopathological mechanisms, constituting an important knowledge base for research on adverse childhood experiences and personality disorders. Overall, the core cited literature and academic influence in this field are mainly concentrated in psychiatry, trauma psychology, and personality pathology.

### 3.9 Analysis of national scientific impact and citation performance

**Figure 9:**
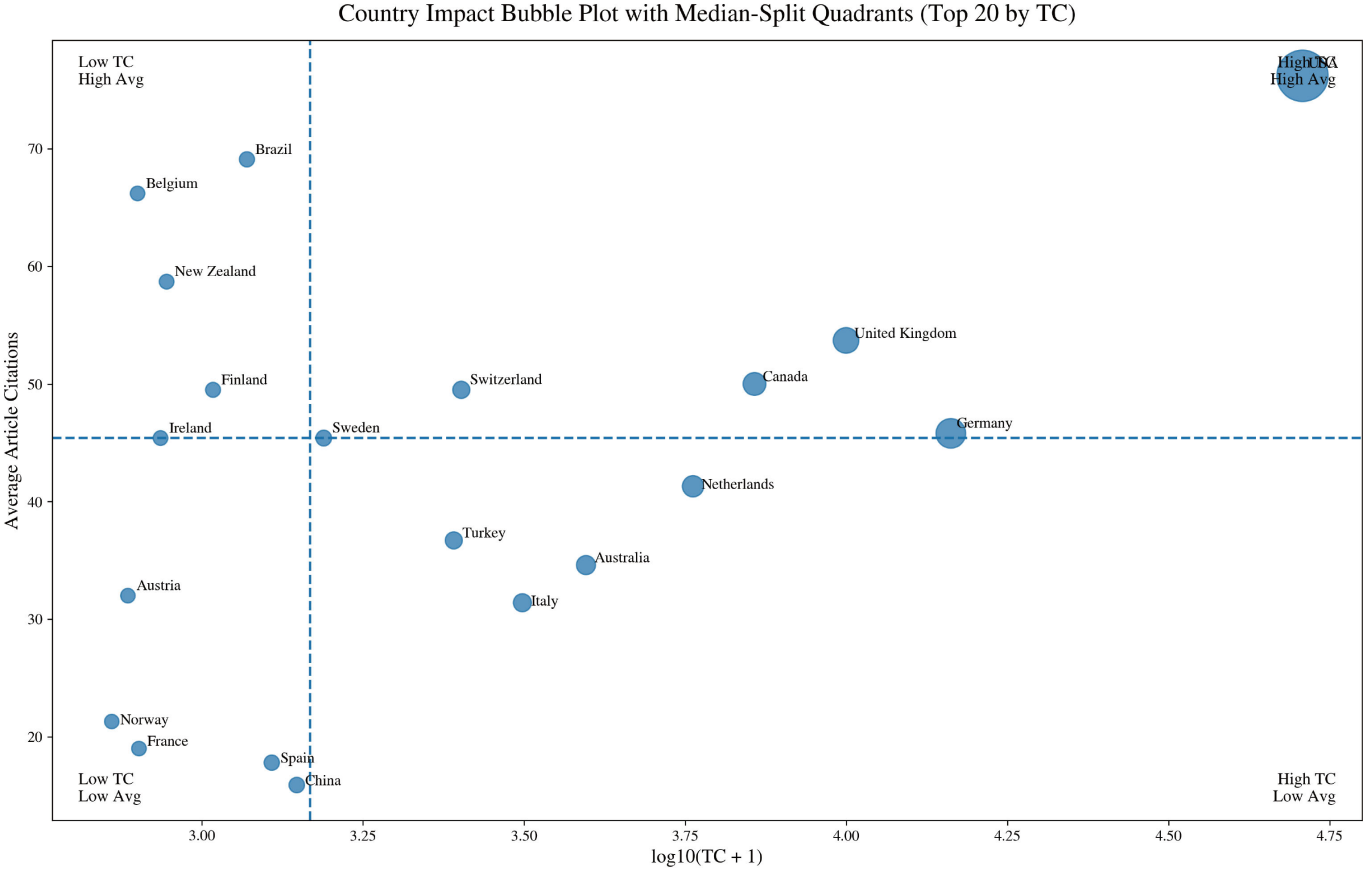
Country impact bubble plot with median-split quadrants (Top 20 by total citations). The x-axis shows log10(TC + 1), where TC denotes total citations, and the y-axis indicates average article citations. Dashed lines represent median splits, dividing countries into four quadrants (high TC/high average, high TC/low average, low TC/high average, low TC/low average). Each bubble represents a country (Top 20 by TC), enabling comparison of overall citation impact versus per-article impact.

As shown in **Figure 9**, the national impact bubble plot further compared the overall citation impact and average citation level per publication among highly cited countries/regions. The x-axis represents the log-transformed total citations [log10(TC + 1)], while the y-axis represents the average number of citations per publication. The dashed lines indicate the median division lines. Overall, the USA was located in the upper-right quadrant and had the largest bubble size, indicating that it not only had the highest overall citation impact but also maintained a high average citation level per publication, making it the most academically influential country in this field. The United Kingdom, Canada, Germany, and Switzerland were also located in or near the upper-right quadrant, suggesting that these countries had strong influence in terms of both total citation volume and average citation performance per publication.

In contrast, the Netherlands, Australia, Italy, and Turkey were located in the lower-right quadrant, indicating that these countries had relatively high overall citation impact but average citation levels per publication below the median, possibly reflecting a relatively large research output with uneven citation impact distribution. Brazil, Belgium, New Zealand, and Finland were located in the upper-left quadrant. Although their total citation counts were relatively low, their average citation levels per publication were high, suggesting that some of their studies had high individual impact. China, Spain, France, Norway, and Austria were mainly distributed in the lower-left quadrant, indicating that there remains room for improvement in both total citation impact and average citation level per publication. Overall, this figure indicates that the USA occupies a clearly dominant position in the field of adverse childhood experiences and personality disorders, while some countries in Europe, North America, and Oceania have also developed relatively stable academic influence.

### 3.10 Global and local citation analysis of ACEs and PDs research

**Table 3** lists the top 30 representative publications ranked by local citations in this field. Overall, these highly cited publications mainly focused on childhood abuse, childhood trauma, borderline personality disorder, post-traumatic stress disorder, emotion dysregulation, and neurobiological mechanisms, indicating that the association between adverse childhood experiences and personality disorders has long been a core knowledge foundation of this field.

**Table 3:** Information on the top 30 literature sorted by lobal citations score.

Number	First author	Article name	source	Year	Lobal citations	Global citations	LC/GC ratio (%)	Normalized Local Citations	Normalized global citations
1	JOHNSON JG	Childhood maltreatment increases risk for personality disorders during early adulthood	ARCH GEN PSYCHIAT	1999	173	581	29.78	12.01	3.68
2	PORTER C,	Childhood adversity and borderline personality disorder: a meta-analysis	ACTA PSYCHIAT SCAND	2020	122	307	39.74	38.11	9.78
3	BALL JS	Borderline personality disorder and childhood trauma: evidence for a causal relationship	CURR PSYCHIAT REP	2009	106	232	45.69	12.45	3.21
4	AFIFI TO	Childhood adversity and personality disorders: results from a nationally representative population-based study	J PSYCHIATR RES	2011	97	298	32.55	10.78	3.64
5	DRIESSEN M	Magnetic resonance imaging volumes of the hippocampus and the amygdala in women with borderline personality disorder and early traumatization	ARCH GEN PSYCHIAT	2000	93	416	22.36	7.02	3.33
6	LEICHSENRING F	Borderline personality disorder	LANCET	2011	80	634	12.62	8.89	7.74
7	YEN S	Traumatic exposure and posttraumatic stress disorder in borderline, schizotypal, avoidant, and obsessive-compulsive personality disorders: findings from the collaborative longitudinal personality disorders study	NERV MENT DIS	2002	79	223	35.43	4.96	1.94
8	BIERER LM	Abuse and neglect in childhood: relationship to personality disorder diagnoses	2003, CNS SPECTRUMS	2003	78	179	43.58	5.19	1.53
9	ZANARINI MC	Severity of reported childhood sexual abuse and its relationship to severity of borderline psychopathology and psychosocial impairment among borderline inpatients	NERV MENT DIS	2002	77	242	31.82	4.83	2.11
10	BANDELOW B	Early traumatic life events, parental attitudes, family history, and birth risk factors in patients with borderline personality disorder and healthy controls	PSYCHIAT RES	2005	70	206	33.98	7.00	2.78
11	SCHMAHL CG	Magnetic resonance imaging of hippocampal and amygdala volume in women with childhood abuse and borderline personality disorder	PSYCHIAT RES-NEUROIM	2003	69	220	31.36	4.59	1.89
12	ANDA RF	The enduring effects of abuse and related adverse experiences in childhood. A convergence of evidence from neurobiology and epidemiology	PSY CLIN N	2006	68	3090	2.20	5.78	18.14
13	BOHUS M	Dialectical behaviour therapy for post-traumatic stress disorder after childhood sexual abuse in patients with and without borderline personality disorder: a randomised controlled trial	PSYCHOTHER PSYCHOSOM	2013	67	233	28.76	8.06	3.03
14	LANIUS RA	Emotion modulation in PTSD: Clinical and neurobiological evidence for a dissociative subtype	AM J PSYCHIAT	2010	63	810	7.78	6.82	6.49
15	PAGURA J	Comorbidity of borderline personality disorder and posttraumatic stress disorder in the U.S. population	J PSYCHIATR RES	2010	63	217	29.03	6.82	1.74
16	CARR CP	The role of early life stress in adult psychiatric disorders: a systematic review according to childhood trauma subtypes	NERV MENT DIS	2013	59	652	9.05	7.10	8.48
17	TEICHER MH	Childhood maltreatment and psychopathology: A case for ecophenotypic variants as clinically and neurobiologically distinct subtypes	AM J PSYCHIAT	2013	59	769	7.67	7.10	10.00
18	GOLIER JA	The relationship of borderline personality disorder to posttraumatic stress disorder and traumatic events	AM J PSYCHIAT	2003	58	159	36.48	3.86	1.36
19	FERREIRA LFD	Borderline personality disorder and sexual abuse: A systematic review	PSYCHIAT RES	2018	58	111	52.25	15.02	3.27
20	DALENBERG CJ	Evaluation of the evidence for the trauma and fantasy models of dissociation	PSYCHOL BULL	2012	56	473	11.84	6.78	3.30
21	TEICHER MH	The effects of childhood maltreatment on brain structure, function and connectivity	NAT REV NEUROSCI	2016	55	1331	4.13	11.50	22.66
22	KUO JR		CHILD ABUSE NEGLECT	2015	54	104	51.92	11.64	2.12
23	TEICHER MH	The neurobiological consequences of early stress and childhood maltreatment	NEUROSCI BIOBEHAV R	2003	52	1055	4.93	3.46	9.04
24	BRAMBILLA P	Anatomical MRI study of borderline personality disorder patients	PSYCHIAT RES-NEUROIM	2004	52	129	40.31	5.15	1.39
25	JOHNSON JG	Childhood verbal abuse and risk for personality disorders during adolescence and early adulthood	COMPR PSYCHIAT	2001	50	168	29.76	5.19	1.95
26	SAR V	Axis I dissociative disorder comorbidity in borderline personality disorder and reports of childhood trauma	J CLIN PSYCHIAT	2006	49	138	35.51	4.17	0.81
27	TYRKA AR	Childhood maltreatment and adult personality disorder symptoms: influence of maltreatment type	PSYCHIAT RES	2009	49	119	41.18	5.76	1.65
28	CLOITRE M	Distinguishing PTSD, Complex PTSD, and Borderline Personality Disorder: A latent class analysis	EUR J PSYCHOTRAUMATO	2014	49	299	16.39	8.16	5.58
29	DANNLOWSKI U	Limbic scars: long-term consequences of childhood maltreatment revealed by functional and structural magnetic resonance imaging	BIOL PSYCHIAT	2012	48	776	6.19	5.81	5.41
30	PERROUD N	Increased methylation of glucocorticoid receptor gene (NR3C1) in adults with a history of childhood maltreatment: a link with the severity and type of trauma	TRANSL PSYCHIAT	2011	47	372	12.63	5.22	4.54

In terms of local citations, the study by Johnson et al. (45), published in Archives of General Psychiatry in 1999 and titled “Childhood maltreatment increases risk for personality disorders during early adulthood,” ranked first, with 173 local citations and 581 global citations. This indicates that the study had a strong knowledge-transmission role within the present dataset (45). The meta-analysis by Porter et al. in 2020 on the relationship between childhood adversity and borderline personality disorder ranked second, with 122 local citations and the highest normalized local citation value of 38.11. Although this article was published relatively recently, it has rapidly become an important integrative publication in this field (15). The review by Ball et al. in 2009 on the causal relationship between borderline personality disorder and childhood trauma ranked third, with 106 local citations and a local/global citation ratio of 45.69%, indicating its strong field-specific influence within the research topic (46).

In terms of global citations, the study by Anda et al. in 2006 on the long-term effects of childhood abuse and related adverse experiences had the highest number of global citations, reaching 3,090. However, its local/global citation ratio was only 2.20%, suggesting that this article had broad interdisciplinary influence but was not limited to the field of personality disorder research (47).

The series of studies by Teicher et al. on the neurobiological consequences of childhood maltreatment also showed high global influence (48). Among them, the article published in Nature Reviews Neuroscience in 2016 had 1,331 global citations and the highest normalized global citation value of 22.66 (27), while the article published in Neuroscience and Biobehavioral Reviews in 2003 had 1,055 global citations. These findings indicate that the long-term effects of childhood trauma on brain structure, function, and connectivity represent an important mechanistic direction for explaining the relationship between childhood adversity and personality pathology (49).

In addition, from the perspective of the local/global citation ratio, the systematic review by Ferreira et al. in 2018 on borderline personality disorder and sexual abuse had the highest ratio, at 52.25% (50). The study by Kuo et al. in 2015 on the relationship among childhood emotional abuse, emotion regulation difficulties, and borderline personality disorder features had a ratio of 51.92%. Although these publications had relatively lower global citation counts, they were frequently cited within the present research topic, suggesting that they more strongly reflect the specialized knowledge structure at the intersection of adverse childhood experiences and personality disorders (51). Overall, the highly cited literature indicates that the research foundation of this field mainly revolves around the knowledge trajectory of “childhood abuse/trauma–borderline personality disorder–PTSD/dissociative symptoms–emotion regulation–neurobiological alterations.”

## 4 Discussion

### 4.1 Principal findings

Using bibliometric methods, this study systematically examined the overall developmental trajectory, international collaboration structure, core journal sources, major research contributors, and thematic evolution of studies on ACEs and PDs. By integrating bibliographic records from the WoSCC, Scopus, and PubMed, this study expanded literature coverage and provided a macro-level overview of annual publication trends, scientific output and collaboration networks across countries/regions, institutions, and authors, as well as keyword co-occurrence structures. Meanwhile, considering that citation relationship analyses and research-frontier identification require complete, standardized, and database-consistent reference fields, this study further conducted co-citation analysis, cluster analysis, and burst term detection based on WoSCC data to reveal the knowledge base and thematic evolution of this field.

After multi-database merging, year screening, document-type screening, and hierarchical deduplication, 5,084 publications were finally included for analysis. Compared with studies based on a single database, the data sources of this study were more comprehensive, allowing the developmental profile of ACEs and PDs research to be reflected on a broader literature basis. The annual publication trend showed that the number of publications in this field was relatively low in the early stage, gradually increased after 2000, and has remained at a high level in recent years. Publication output reached a recent peak in 2023 and remained high in 2025. This trend suggests that the relationship between adverse childhood experiences and personality disorders has become an important topic of sustained interest in psychiatry, psychology, and public health.

Previous narrative reviews, systematic reviews, meta-analyses, and umbrella reviews have mainly focused on clarifying the associations between adverse childhood experiences and different types of personality disorders, particularly specific diagnoses or clusters such as borderline personality disorder and Cluster C personality disorders (15, 45, 46, 52). In contrast, the present study further extends the existing evidence from a macro-level knowledge-mapping perspective by systematically presenting annual publication trends, country and institutional contributions, international collaboration patterns, journal distribution, core authors, keyword structures, thematic evolution, and citation network characteristics. Therefore, this study does not replace previous clinical or epidemiological reviews; rather, it complements them by illustrating how this field has developed at the level of literature structure and research evolution, which research forces have driven it, and which directions it may take in the future.

Overall, the findings suggest that ACEs–PDs research has gradually shifted from an early focus on “exposure–outcome” associations toward deeper issues such as mechanistic explanations, clinical phenotypes, comorbidity patterns, and intervention translation (26, 27, 53, 54). This shift was also reflected in the keyword and burst term analyses. Earlier studies were more centered on diagnostic classification, childhood abuse, personality disorders, and psychiatric comorbidity, whereas recent themes such as emotion dysregulation, social support, early intervention, functional connectivity, non-suicidal self-injury, and mental health have become increasingly prominent. These findings indicate that the field is extending from simple association studies toward mechanistic research, risk stratification, and translational applications (26, 55, 56). This transition not only reflects the deepening of research questions but also suggests that future studies need to further integrate psychosocial mechanisms, neurobiological evidence, and clinical intervention pathways to more comprehensively explain how childhood adversity influences the developmental process of personality pathology.

### 4.2 Research growth trends and disciplinary driving factors

The annual publication trend showed that research on adverse childhood experiences and personality disorders has generally progressed from a slow-starting phase to rapid growth and then to sustained activity. The limited number of publications in the early stage indicates that this topic had not yet formed a stable academic field. As concepts such as childhood trauma, attachment impairment, developmental psychopathology, and trauma-related personality pathology gradually gained attention, the number of related publications began to increase steadily. The marked rise in publication output after 2000 may be related to the promotion of the ACEs theoretical framework, the development of trauma-informed mental health research, and growing attention to clinical issues such as borderline personality disorder, emotion dysregulation, self-injurious behavior, and interpersonal dysfunction.

In recent years, this field has continued to maintain a high publication level, indicating that the relationship between adverse childhood experiences and personality disorders remains an active research direction. Notably, although association studies remain important, the focus of the field appears to be shifting from “whether childhood adversity increases the risk of personality disorders” to “how specific types, timing, duration, and severity of childhood adversity influence personality pathology through emotion regulation, attachment patterns, stress-response systems, neurodevelopment, and dimensional personality traits (26, 53, 54, 57).” This shift helps connect epidemiological evidence with clinical assessment, prevention, and intervention strategies.

First, the conceptual framework of adverse childhood experiences and its public health significance for lifelong health risks have received increasing attention. This has transformed early adversity from merely an “individual experience” into a quantifiable and potentially modifiable risk exposure (14, 58, 59). A systematic review and meta-analysis by Hughes et al. published in The Lancet Public Health reported stable associations between multiple ACEs and a wide range of health outcomes, and further suggested that these outcomes may increase adversity exposure in the next generation through pathways such as violence, mental illness, and substance misuse, implying potential intergenerational cumulative effects. Therefore, evidence-based primary prevention of ACEs and risk-buffering strategies, such as strengthening household economic support, promoting protective social norms, improving parenting and early developmental support, and providing supportive services for individuals who have already experienced adversity, are considered important directions for reducing the lifelong health burden of ACEs (14, 60). Meanwhile, trauma-informed service systems can provide an organizational and practical framework for identifying and responding to adversity-related needs (61). Therefore, evidence-based primary prevention of ACEs and risk-buffering strategies, such as strengthening household economic support, promoting protective social norms, improving parenting and early developmental support, and providing supportive services for individuals who have already experienced adversity, are considered important directions for reducing the lifelong health burden of ACEs. Meanwhile, trauma-informed service systems can provide an organizational and practical framework for identifying and responding to adversity-related needs. From a broader perspective, such cross-sectoral actions may also align with the goals of promoting health and preventing violence advocated in the United Nations 2030 Sustainable Development Goals (SDGs), thereby providing a global platform for policy coordination and resource integration (61–64).

Second, advances in developmental psychopathology and dimensional models of psychopathology have greatly promoted longitudinal research on the mechanisms and developmental trajectories of personality pathology, leading to two key paradigm shifts. First, within the developmental psychopathology framework, personality pathology is viewed as a dynamic process that changes across developmental stages and is jointly driven by risk exposures, such as childhood adversity, individual vulnerabilities, such as temperament and genetics, and environmental feedback, such as parenting quality, peer relationships, and social support. This perspective emphasizes the differential effects of developmental cascades and “timing × chronicity” on later psychopathology (65–68). Accordingly, research no longer focuses solely on comparisons of “disease presence versus absence,” but increasingly relies on longitudinal cohorts and advanced statistical models to describe symptom onset, growth rate, fluctuation patterns, and critical turning points, while testing stage-specific sensitivity and the cumulative effects of adversity exposure. For example, some longitudinal studies have directly examined the effects of the timing and duration of childhood maltreatment on subsequent psychopathological development, whereas others have investigated the differential predictive roles of abuse and neglect in later psychopathological trajectories under dimensional models of adversity, such as the threat/deprivation framework. Trajectory-based studies have also revealed developmental chains in adolescent samples, such as “childhood maltreatment–peer bullying–borderline personality features–suicide risk” (69, 70). Second, dimensional models encourage researchers to decompose personality pathology into continuous dimensions of impaired personality functioning, involving self and interpersonal functioning, together with pathological traits and process mechanisms. This approach is more suitable for capturing heterogeneity through longitudinal research methods. Researchers may use latent growth models and latent class/profile analyses to identify different developmental subtypes, such as persistently high-risk, late-onset increasing, and recovery/remission trajectories. Cross-lagged or longitudinal mediation frameworks can also be used to examine the temporal sequence and potential causal chain of “childhood adversity → process phenotypes, such as emotion regulation, impulse control, and social information processing → personality dysfunction.” In this context, transdiagnostic dimensional frameworks such as HiTOP are considered helpful for addressing the limitations of traditional categorical diagnoses in explaining heterogeneity and comorbidity, while also providing more stable phenotypic targets for longitudinal mechanism research and clinical neuroscience. At the same time, dimensional reforms in personality disorder diagnostic systems, such as the ICD-11 personality disorder model, have further promoted severity- and trait-domain-centered assessment and research designs, and strengthened conceptual and measurement mapping with the Alternative Model for Personality Disorders in DSM-5 (71–73).

Third, the rapid development and increasing accessibility of neuroimaging, genetic/epigenetic, and stress biology methods have promoted a shift in this field from early “epidemiological association description” toward “neurobiological mechanism explanation” and “cross-level evidence-chain validation.” At the neuroimaging level, increasing numbers of studies have used task-based functional magnetic resonance imaging (fMRI), resting-state functional connectivity (rsFC), structural magnetic resonance imaging (MRI), and more systematic evidence synthesis methods to characterize neural phenotypes associated with early adversity exposure. Classic neurobiological reviews have shown that childhood abuse and neglect may exert lasting effects on multiple brain systems, particularly regions and pathways related to threat processing, emotion regulation, and reward sensitivity, including the amygdala, prefrontal–limbic regulatory circuits, and striatal reward systems. These findings provide a potential neurobiological basis for core dimensions of personality pathology such as emotional instability, impulsivity, interpersonal sensitivity, and maladaptive social cognition (27, 74, 75).

Previous population-based studies, reviews, and meta-analyses have also consistently supported an association between childhood adversity and personality pathology, especially the relatively clear relationship with borderline personality disorder. At the same time, childhood adversity may also function as a transdiagnostic risk factor influencing the onset and development of different personality disorder categories (8, 15, 17). Consistent with these studies, the bibliometric findings of the present study further show that the research focus in the ACEs–PDs field has gradually shifted from early “exposure–outcome” association descriptions toward directions that emphasize mechanistic explanation and clinical translation, including emotion dysregulation, neurobiological vulnerability, developmental timing, risk stratification, and clinically meaningful intervention targets. More recent evidence further suggests that these effects may manifest as alterations in resting-state brain network organization and amygdala-related connectivity patterns. A systematic review of resting-state functional connectivity in maltreated/neglected adolescents showed that reproducible network-level findings are gradually emerging, particularly involving altered coupling between the amygdala and key networks such as the default mode network and salience network (76). Meta-analytic evidence across task domains also supports a long-term association between a history of severe adversity and enhanced adult amygdala responses to various psychological challenges, as well as reduced prefrontal cortex responses (77). At the molecular and cross-mechanistic levels, research has also progressed from “candidate biological pathway speculation” toward more direct biomarker evidence. On the one hand, DNA methylation of stress-related genes, such as NR3C1, and negative feedback regulation of the hypothalamic–pituitary–adrenal (HPA) axis have been repeatedly proposed as key pathways. Reviews on NR3C1 methylation and cortisol/HPA-axis markers have summarized relatively consistent associations between childhood trauma exposure and NR3C1 epigenetic alterations, and emphasized their potential links with dysregulated stress-response regulation (32, 78). On the other hand, inflammatory and immune-related pathways are increasingly regarded as important bridges linking “early adversity–psychopathology–physical health risk” (79). As described by Danese et al., childhood trauma is an important risk factor for psychopathology, but its biological translational pathways remain incompletely understood (55, 79). Human studies and animal models suggest that early developmental stress can induce persistent systemic inflammatory responses and may alter the function of cognitive and emotional systems, as well as responses to subsequent stress, by affecting neurodevelopment and brain network maturation, thereby increasing the risk of psychopathology (26, 27, 55). More longitudinal and cross-level mechanistic studies are needed to clarify the key mediating links and intervention targets in the “childhood trauma–inflammatory pathway–psychopathology” pathway. Overall, integrating multimodal imaging, epigenetics, and immune/stress biomarkers is facilitating the construction of a cross-level evidence chain of “adverse childhood experiences → neural and stress-immune abnormalities → process phenotypes → personality dysfunction and personality pathology,” thereby improving the falsifiability and translational value of mechanistic hypotheses.

### 4.3 Country/regional landscape and international collaboration: centralized and differentiated collaboration patterns

Country/region analysis showed that the United States occupies a clearly dominant position in research on adverse childhood experiences and personality disorders. It not only had the highest publication output but also occupied a central position in both the international collaboration network and the national impact bubble plot. This indicates that the United States has important influence in agenda setting, theoretical framework construction, empirical evidence accumulation, and academic dissemination in this field. The United Kingdom, Germany, Canada, the Netherlands, Australia, and Switzerland also showed high scientific output and active collaboration, jointly forming the main international research backbone of this field. Overall, this field has formed a collaboration network centered on North American and European countries, but there are still marked imbalances in participation and collaboration intensity across different countries/regions.

In terms of collaboration patterns, Switzerland, Spain, the United Kingdom, the Netherlands, and Germany had relatively high proportions of multiple-country publications (MCPs), suggesting that these countries are more inclined to engage in cross-national collaboration. The dense collaborative links among European countries may be related to long-standing regional academic collaboration traditions, transnational research networks, and the institutional design of European multinational funding programs. For example, some European research projects usually encourage or require institutions from different countries to participate jointly, which objectively promotes cross-national collaboration and co-authorship (80). By contrast, although the United States occupies a central position in the collaboration network, its large domestic research scale, rich sample resources, and mature medical and scientific research systems may allow it to complete a large number of high-quality studies through domestic networks to some extent. Therefore, its proportion of international collaboration does not necessarily correspond directly to its academic influence. In addition, some funding and compliance frameworks have relatively clear definitions and management requirements for cross-border collaboration or “foreign components,” which may increase the organizational and administrative costs of international collaboration and, to some extent, encourage research teams to rely more on domestic research networks for core research procedures (81, 82).Thus, MCP and SCP should not be interpreted simply as indicators of research quality. Instead, they should be understood comprehensively in relation to national research scale, research resources, funding models, and academic dissemination systems.

The national impact bubble plot further supplemented these findings. The United States had both the highest total citations and a high average citation level per publication, indicating that it not only had a large output scale but also strong overall academic influence. The United Kingdom, Canada, Germany, and Switzerland also showed strong competitiveness in terms of total citation impact and average citation performance per publication. In contrast, some countries had relatively low total citation counts but high average citations per publication, such as Brazil, Belgium, New Zealand, and Finland. This may reflect that some of their studies focused on high-impact topics or achieved higher citation returns per publication through international collaboration and high-visibility journals. Previous bibliometric studies have also indicated that international collaboration is usually associated with higher citation performance, possibly due to methodological complementarity across multinational teams, higher journal visibility, and broader dissemination networks (83). Conversely, China, Spain, France, Norway, and Austria showed relatively lower total citation impact and average citation levels per publication in the current dataset, suggesting that there remains room for improvement in international visibility, high-impact output, and depth of international collaboration.

It should be noted that citation impact cannot be directly equated with research quality. Citation performance across countries may be influenced by multiple factors, including publication language, journal selection, degree of international collaboration, popularity of research topics, time since publication, and database coverage. In particular, because this study included only English-language publications, it may underestimate the research contributions of non-English-speaking countries and regions. For China and other Asian countries, some relevant studies may have been published in local-language journals or may not have been fully indexed in international databases. Therefore, the national distribution and impact pattern presented in this study more accurately reflect research visibility in international English-language databases rather than the complete global distribution of all relevant studies. Moreover, previous studies have shown that different databases have systematic differences in journal coverage, disciplinary emphasis, and indexing standards, which may alter the statistical output set and affect the robustness of cross-national comparisons (36, 84).

Overall, this field has formed an international collaboration pattern centered on the United States, with the United Kingdom, Germany, Canada, the Netherlands, Australia, and Switzerland serving as important nodes. Future studies should further strengthen collaboration between core research countries and regions such as Asia, South America, and Africa, and promote the development of cross-cultural samples, standardized measurement tools, and multicenter longitudinal studies, thereby improving the global representativeness and external validity of evidence in research on adverse childhood experiences and personality disorders.

### 4.4 Journal ecology: coexistence of high-output publication platforms and high-impact knowledge sources

The journal-level results indicate that ACEs–PDs research has formed a relatively clear dual structure consisting of “publication platforms” and “knowledge-source journals.” First, **Table 2** shows that the high-output source journals in this field are mainly concentrated in psychiatry, personality disorders, child abuse and neglect, interpersonal violence, affective disorders, and developmental psychopathology, suggesting that ACEs–PDs research has obvious interdisciplinary characteristics. Among them, general psychiatry journals such as Psychiatry Research, Journal of Nervous and Mental Disease, and Comprehensive Psychiatry published relevant studies relatively early and have continued to do so, indicating that the relationship between ACEs and personality disorders has long been regarded as an important issue in psychiatry and clinical psychology. The Journal of Personality Disorders, as a specialist journal in the field of personality disorders, ranked high in publication output, suggesting sustained attention within the personality disorder field to the role of childhood adversity in the development of different forms of personality pathology. Meanwhile, the presence of journals such as Journal of Interpersonal Violence and Child Abuse & Neglect indicates that trauma exposure, violent experiences, abuse, and neglect constitute important entry points for ACEs–PDs research. Development and Psychopathology further reflects the theoretical value of the developmental psychopathology framework in explaining how early environmental risks influence personality structure and the formation of psychopathology.

On the other hand, the highly cited sources shown in **Figure 8A** further suggest that the knowledge base of this field mainly originates from journals related to psychiatry, personality disorders, child abuse and trauma, clinical psychology, and developmental psychopathology. General psychiatry and clinical psychology journals such as American Journal of Psychiatry, Biological Psychiatry, Archives of General Psychiatry, Psychological Medicine, and British Journal of Psychiatry occupied important positions in the co-citation network, indicating that theoretical frameworks, classic cohort studies, diagnostic systems, systematic reviews, and mechanistic evidence in this field often come from high-impact general journals. At the same time, journals such as Child Abuse & Neglect, Journal of Traumatic Stress, Development and Psychopathology, and Clinical Psychology Review also constitute important knowledge sources, reflecting the close connections among childhood adversity, trauma exposure, developmental psychopathology, and personality pathology research.

Overall, the journal ecology of this field shows clear interdisciplinary features: high-output journals provide stable publication channels for ACEs–PDs research, whereas highly cited source journals constitute the important knowledge base and theoretical support of the field. This “dual structure” indicates that ACEs and PDs research depends not only on the sustained accumulation of specialized fields such as personality disorders, trauma, interpersonal violence, and child abuse, but also on the core theories and methods of psychiatry, developmental psychopathology, and clinical psychology. In addition, possible formatting differences in source journal names, such as “Child Abuse and Neglect” and “Child Abuse & Neglect,” also suggest that future bibliometric studies should further strengthen journal name standardization to avoid splitting the same journal into multiple sources, which may affect statistical accuracy and interpretation of network structures (85).

### 4.5 Institutional and author ecology: concentration of key contributors, cross-institutional collaboration, and differences between productivity and impact

The institutional and author-level results further reveal a relatively concentrated academic ecology in the ACEs–PDs research field. At the institutional level, highly productive institutions were mainly concentrated in comprehensive universities, psychiatric research institutions, and clinical medical centers in Europe and North America. Harvard University had the highest publication output, followed by the University of London and Ruprecht Karls University Heidelberg, suggesting that high-level medical and psychological research institutions in the United States and Europe occupy important positions in this field. Institutions such as King’s College London, Harvard University Medical Affiliates, University College London, the Central Institute of Mental Health, and McLean Hospital also showed high output, indicating that the sustained development of this field depends on the joint promotion of multidisciplinary platforms involving psychiatry, psychology, medicine, and public health.

This institutional distribution indicates that ACEs–PDs research has clear medicine–psychology interdisciplinary characteristics. Comprehensive universities usually have advantages in theoretical construction, epidemiological research, public health perspectives, and interdisciplinary integration, whereas psychiatric research centers, affiliated hospitals, and specialized medical institutions are more likely to accumulate clinical samples related to personality disorders, childhood trauma, affective disorders, dissociative symptoms, and comorbid psychiatric disorders. Therefore, the concentrated distribution of highly productive institutions may reflect the important support provided by long-term stable research teams, clinical resources, longitudinal cohort data, and interdisciplinary collaboration platforms for research output in this field.

It should be noted that institution-level bibliometric results are influenced to some extent by database rules for institutional name standardization and merging. For example, the simultaneous appearance of Harvard University and Harvard University Medical Affiliates among highly productive institutions suggests that system-level universities, affiliated hospitals, medical centers, and research institutes may be incompletely split or merged. Therefore, institutional rankings should be interpreted as output distributions under database indexing rules rather than as precise rankings of the true scientific capacity of individual institutions.

The author-level results similarly showed a core group of researchers. Zanarini M had the highest publication output, followed by Fonagy P, Schmahl C, Paris J, Kleindienst N, Ross C, Bohus M, Arntz A, and Sar V. These authors have long been active in research areas related to borderline personality disorder, childhood trauma, attachment, dissociation, emotion regulation, mechanisms of personality pathology, and psychotherapy, reflecting the joint influence of clinical psychiatry, trauma psychology, and personality disorder research traditions on ACEs–PDs research.

At the same time, authors with higher publication output are not necessarily identical to authors with the highest co-citation influence. Publication output mainly reflects a researcher’s sustained productivity in the field, whereas co-citation frequency more strongly reflects the influence of their work on the knowledge base, theoretical frameworks, or methodological pathways. Some authors may occupy important positions in the knowledge structure of this field through key cohort studies, classic theoretical models, diagnostic studies, mechanistic research, or high-quality reviews. Therefore, interpreting author publication output together with co-cited author results helps distinguish between the two different dimensions of contribution: “research productivity” and “academic influence.”

Overall, the institutional and author-level findings jointly indicate that ACEs–PDs research has formed an academic structure driven by a small number of core institutions and core authors, while also being continuously participated in by a broader research community. This structure is conducive to the formation of stable research directions and the cumulative development of an evidence base, but it may also lead to the concentration of research topics, sample sources, and theoretical perspectives among a limited number of high-output teams or institutions in Europe and North America. Future research should further strengthen collaboration across countries, institutions, and disciplinary teams, especially by promoting multicenter longitudinal cohorts, cross-cultural sample comparisons, standardized measurement tools, and data-sharing mechanisms, thereby improving the representativeness, comparability, and reproducibility of research findings.

### 4.6 Thematic structure: from “exposure–outcome association” to a multilevel evidence chain of “mechanism–translation”

Keyword analysis and citation analysis jointly indicate that research on adverse childhood experiences and personality disorders has formed a relatively stable thematic structure. Its core trajectory mainly revolves around “childhood abuse/trauma–personality disorders, especially borderline personality disorder–psychiatric comorbidity–emotion regulation and neurobiological mechanisms.” Keyword frequency results based on the merged dataset from WoSCC, Scopus, and PubMed showed that terms such as “childhood adversity,” “personality disorder,” “borderline personality disorder,” “depression,” “childhood sexual abuse,” and “post-traumatic stress disorder” were particularly prominent. This suggests that the field has long focused on the relationships among adverse childhood experiences, personality pathology, post-traumatic stress, depressive symptoms, and related psychiatric and psychological outcomes. Meanwhile, keywords such as “antisocial personality disorder,” “anxiety,” “comorbidity,” “self-harm and suicidality,” “aggression,” “violence exposure,” “substance use disorder,” “psychotherapy,” and “trauma” also showed high frequencies, indicating that this field does not focus only on a single personality disorder diagnosis but has gradually developed a multidimensional research framework covering psychiatric comorbidity, behavioral risk, and clinical intervention.

The VOSviewer keyword co-occurrence network further supports these findings. **Figure 6** shows that keywords such as “personality disorder,” “borderline personality disorder,” “childhood sexual abuse,” “child abuse,” “depression,” “post-traumatic stress disorder,” “anxiety,” and “antisocial personality disorder” are located in the core area of the network, indicating that the links among early adversity exposure, personality disorder diagnosis, and related psychiatric symptoms constitute the most stable research foundation of this field. Multiple studies have shown that adverse childhood experiences are closely associated with the occurrence, symptom severity, and clinical complexity of borderline personality disorder (15, 46, 86). Newnham et al. suggested that adolescence is a critical window for personality development, and trauma exposure before or during adolescence, particularly sexual abuse, may have more pronounced adverse effects on personality development and further increase the risk of borderline personality disorder (87). Therefore, the central position of borderline personality disorder in the keyword network reflects not only its high level of attention in clinical research but also its potential role as an important research node connecting childhood adversity, emotion dysregulation, self-injury risk, and complex comorbidity.

In terms of thematic content, three interrelated research levels can be broadly identified in this field. The first level concerns the association between exposure types and personality pathology. High-frequency keywords such as “childhood sexual abuse,” “child abuse,” “childhood adversity,” and “trauma” indicate that researchers have continuously focused on the differential associations between various types of childhood adversity, including abuse, neglect, family dysfunction, and violence exposure, and different personality disorder phenotypes. This is consistent with the core conclusions of locally highly cited publications, such as Johnson et al.’s study on childhood maltreatment increasing the risk of personality disorders in early adulthood and Porter et al.’s (15) meta-analysis on the relationship between childhood adversity and borderline personality disorder, both of which indicate that exposure–outcome associations remain an important component of the knowledge base of this field (15, 45).

The second level involves intermediate phenotypes and the spectrum of psychiatric comorbidity. The presence of keywords such as “depression,” “anxiety,” “post-traumatic stress disorder,” “comorbidity,” “self-harm and suicidality,” “dissociation,” and “substance use disorder” in the keyword network indicates that personality disorders are not isolated outcomes, but are often intertwined with post-traumatic symptoms, mood disorders, anxiety disorders, dissociative symptoms, self-injury/suicide risk, and substance use problems (65). Research at this level usually focuses on how adverse childhood experiences promote the formation and maintenance of personality pathology through process phenotypes such as emotion regulation difficulties, negative affect, impulsivity, interpersonal sensitivity, and trauma-related symptoms (56, 88). The recent activity of themes such as non-suicidal self-injury, emotion dysregulation, and social support also suggests that the field is moving beyond simple descriptions of risk associations toward clinical risk identification and exploration of intervention targets (88, 89). Calvo et al.’s systematic review and meta-analysis on childhood maltreatment and adolescent non-suicidal self-injury also supports this direction (56, 88), indicating that childhood maltreatment, especially emotional and/or sexual abuse, may increase the risk of non-suicidal self-injury in adolescents (66).

The third level involves neurobiological and cross-level mechanism validation. The CiteSpace results in **Figure 7** show that keywords such as “functional connectivity,” “early life stress,” “impulsivity,” “psychotic symptoms,” and “nonsuicidal self injury” exhibited bursts during different periods, suggesting that research hotspots in this field have gradually expanded toward neuropsychological mechanisms, brain functional connectivity, impulsivity, self-injurious behavior, and psychotic symptoms (49). Combined with locally highly cited studies on MRI, the HPA axis, and neurobiological mechanisms, especially the series of studies by Teicher et al. (27). on the effects of childhood maltreatment on brain structure, function, and connectivity, it can be seen that “early trauma–altered brain development–abnormal emotional and behavioral regulation” has become an important mechanistic pathway for explaining the relationship between childhood adversity and personality pathology (26, 37). Such mechanistic studies usually have stronger interdisciplinary influence, serving not only theory construction within the personality disorder field but also being widely cited in trauma psychiatry, neuroscience, and public health research.

The CiteSpace analysis based on WoSCC data in **Figure 7** can also be regarded as a supplement and sensitivity validation of the keyword results from the merged three-database dataset. Timeline clustering showed that themes such as “antisocial personality disorder,” “eating disorder,” “adverse childhood experience,” “child maltreatment,” “dysfunctional parenting,” “case report,” “dissociative identity disorder,” and “emotion dysregulation” constituted important clusters in this field. These themes were generally consistent with the high-frequency keywords and VOSviewer co-occurrence network identified in the merged three-database analysis, indicating that the core hotspots of this field were not driven by a single database and showed good stability. Burst term detection further showed that during 2015–2025, keywords such as “major depression,” “DSM-IV,” “axis-i,” “multiple personality disorder,” “antisocial personality disorder,” “psychiatric comorbidity,” and “childhood abuse” had strong burst intensity, reflecting that earlier research paid greater attention to diagnostic classification, personality disorder types, childhood abuse, and psychiatric comorbidity. Subsequently, keywords such as “early life stress,” “posttraumatic stress disorder,” “impulsivity,” “psychotic symptoms,” “functional connectivity,” and “nonsuicidal self injury” emerged successively, suggesting that research gradually extended toward early-life stress, neuropsychological mechanisms, impulsivity, psychotic symptoms, and non-suicidal self-injury. Keywords that have remained in a burst state in recent years, such as “social support,” “mental disorders,” “disorder,” “version,” and “difficulty,” indicate that current research is increasingly concerned with social support, psychiatric comorbidity, updates to diagnostic tools, and the complexity of clinical identification and intervention (90, 91).

Local and global citation analyses further revealed the knowledge base of this field. **Table 3** shows that locally highly cited publications were mainly concentrated in areas such as childhood abuse, childhood trauma, borderline personality disorder, post-traumatic stress disorder, emotion dysregulation, and neurobiological mechanisms. Among them, Johnson et al.’s (45) 1999 study on childhood maltreatment increasing the risk of personality disorders in early adulthood had the highest number of local citations, suggesting that this study plays an important role in knowledge transmission within this field (34).Porter et al.’s 2020 meta-analysis on the relationship between childhood adversity and borderline personality disorder was published more recently but had a high normalized local citation count, indicating that it has rapidly become important integrative evidence in this field (15). Ball et al.’s review on the causal relationship between borderline personality disorder and childhood trauma also had a high local citation proportion, suggesting strong field-specific influence within this interdisciplinary topic (46).

At the same time, some publications had relatively low local citation proportions but high global citation counts, indicating broader interdisciplinary influence (58). For example, Anda et al.’s study on the long-term effects of childhood abuse and related adverse experiences had the highest global citation count, suggesting that ACEs research influences not only the personality disorder field but also public health, psychiatry, and chronic disease research more broadly (14, 47). Teicher et al.’s neurobiological studies on the effects of childhood maltreatment on brain structure, function, and connectivity also showed high global influence (27), indicating that the neurodevelopmental consequences of childhood trauma constitute an important mechanistic direction for explaining the relationship between ACEs and personality pathology (27, 92). In addition, Ferreira et al.’s systematic review on borderline personality disorder and sexual abuse, and Kuo et al.’s study on the relationship among childhood emotional abuse, emotion regulation difficulties, and borderline personality disorder features, did not have the highest global citation counts (50, 93), but had relatively high local/global citation ratios, suggesting that these studies have strong specialized influence within the ACEs–PDs intersection (50, 51).

Overall, keyword analysis, citation analysis, and WoSCC-based CiteSpace results mutually support each other, indicating that research on adverse childhood experiences and personality disorders has formed a relatively stable thematic structure and shows an evolutionary trend from “exposure–outcome association” toward mechanistic explanation, risk identification, and clinical translation. Early research mainly focused on the relationship between childhood abuse/trauma and the risk of personality disorders, especially borderline personality disorder, psychiatric comorbidity, and trauma-related symptoms. In recent years, research hotspots have further extended toward emotion regulation, non-suicidal self-injury, social support, functional connectivity, neurobiological mechanisms, and early intervention. This evolutionary pathway is broadly consistent with the overall development of ACEs research from descriptive association to mechanistic interpretation, and then to risk stratification and intervention translation. Future studies should further strengthen longitudinal cohorts, multimodal neurobiological indicators, inflammatory and epigenetic markers, standardized clinical assessments, and cross-cultural sample validation, so as to more clearly explain how different ACE types, timing, duration, and severity influence different dimensions of personality pathology and provide more actionable evidence for clinical screening, risk prediction, and early intervention.

### 4.7 Knowledge base and key literature: “dual-channel influence” revealed by local/global citations and normalized indicators

Based on the WoSCC sample, this study further compared differences between local citations (LC) and global citations (GC) to reveal different levels of the knowledge base in this field. Overall, publications with high LC rankings mostly focused directly on the core proposition that “childhood maltreatment/adversity increases the risk of personality disorders,” such as Johnson et al.’s classic epidemiological study on childhood maltreatment and the risk of personality disorders in early adulthood, Porter et al.’s meta-analysis on the relationship between childhood adversity and borderline personality disorder, and Ball et al.’s review on the relationship between borderline personality disorder and childhood trauma (15, 45, 46). These publications had high local citation counts within the present corpus, indicating that they are frequently used as theoretical foundations or evidence support in the specific ACEs–PDs research field and constitute key nodes in the internal knowledge structure of this field.

In contrast, some publications had relatively low local citation proportions within the present topic but high global citation impact. For example, Anda et al.’s study on the long-term health effects of adverse childhood experiences and Teicher et al.’s series of studies on the neurobiological consequences of childhood maltreatment (27, 48, 49) have broad influence across multiple fields, including public health, trauma psychiatry, neuroscience, and developmental psychopathology (27, 49, 58, 59). This suggests that GC more readily reflects foundational literature with broad interdisciplinary dissemination and strong overall academic influence, whereas LC is more suitable for identifying core publications that are continuously inherited and repeatedly used within a specific research field.

Therefore, combining LC and GC helps distinguish two different types of knowledge sources: one consists of field-specific core publications that directly promote the development of ACEs–PDs research, and the other consists of foundational publications that provide theoretical background, mechanistic explanation, or interdisciplinary evidence for the field. Overall, highly cited publications show that the knowledge trajectory of research on adverse childhood experiences and personality disorders mainly revolves around “childhood abuse/trauma–borderline personality disorder–PTSD/dissociative symptoms–emotion regulation–neurobiological alterations.” This structure also corroborates the results of keyword co-occurrence and burst term analyses, indicating that the field has gradually expanded from early risk association studies toward psychiatric comorbidity, intermediate phenotypes, and neurobiological mechanistic explanations.

### 4.8 Methodological strengths, limitations, and future directions

An important strength of this study is the use of a hierarchical data integration and analytical strategy. At the macro level, including annual publication trends, country/region contributions, institutional and author output, and keyword co-occurrence network analysis, this study integrated three databases—WoSCC, Scopus, and PubMed—to broaden literature coverage and improve the representativeness of the results. At the level of citation relationship analysis and research-frontier identification, this study conducted co-citation analysis, cluster analysis, and burst detection based on WoSCC data to ensure the completeness of reference fields, consistency of citation-counting rules, and reproducibility of CiteSpace analysis, thereby reducing systematic bias caused by differences in citation coverage, reference formats, and indexing rules across databases. In addition, this study reported local citations (LC), global citations (GC), the LC/GC ratio, and normalized citation indicators, which helped identify core literature and the knowledge base of this field more comprehensively from the perspectives of both “within-field knowledge inheritance” and “interdisciplinary visibility.”

However, this study still has several limitations. First, only English-language publications were included, which may underestimate the research contributions of non-English-speaking countries and regions. This limitation may particularly affect the representation of research from Asia, because some countries, including China, may have relevant studies published in local-language journals. Second, although this study used DOI matching, title verification, and hierarchical deduplication strategies to merge and clean multi-database records, a small number of residual duplicates or record inconsistencies may still remain because different databases vary in their records of titles, authors, institutions, and journal names. Third, differences in institutional and journal name standardization may affect the accuracy of rankings and network structures. For example, different affiliated units, university systems, or medical centers of the same institution may be split or merged across different databases. Fourth, although keyword analysis was cleaned and merged, it may still not fully cover synonyms, abbreviations, and conceptual variants, leading to underestimation or fragmentation of some themes. Fifth, citation analysis was based only on WoSCC. Although this choice improved the methodological consistency and reproducibility of citation network analysis, it may have omitted high-impact publications indexed in Scopus or PubMed but not covered by WoSCC. Meanwhile, because WoSCC has relatively strong coverage of earlier, highly cited, and Western English-language journal literature, WoSCC-based citation analysis may to some extent reinforce the visibility of early classic literature and research from Europe and North America. Sixth, although the search strategy of this study attempted to cover different types of personality disorders and related expressions of personality pathology, the existing literature structure shows that research related to borderline personality disorder and Cluster B personality disorders accounts for a large proportion of this field. Therefore, the findings of this study may more fully reflect the research trajectory related to BPD/Cluster B personality disorders, whereas evidence related to Cluster A and Cluster C personality disorders may be relatively underrepresented. Finally, bibliometric methods mainly reflect research output, academic visibility, collaboration relationships, and thematic evolution trends. They cannot directly evaluate the methodological quality of individual studies, nor can they prove a causal relationship between adverse childhood experiences and personality disorders. Therefore, the results of this study should be understood as a macro-level depiction of the knowledge structure and research trends in this field, rather than as direct validation of clinical effect sizes or causal mechanisms.

Based on the hotspot evolution pathway identified in this study, future research can be advanced in several directions. First, more high-quality longitudinal cohort studies and cross-cultural studies are needed to clarify the differential effects of ACE type, exposure timing, duration, severity, and cumulative burden on the development of personality pathology. Future studies should not focus only on categorical diagnoses of personality disorders, but should also incorporate dimensional personality pathology features such as impaired personality functioning, negative affectivity, impulsivity, interpersonal difficulties, and identity disturbance, thereby better aligning with the current shift in personality disorder research from categorical diagnosis to dimensional models. Second, more attention should be paid to relatively understudied personality disorder types and clusters, especially Cluster A and Cluster C personality disorders, and the specific effects of different ACE subtypes on different personality disorder phenotypes should be further compared. Third, integrated modeling of multimodal mechanistic chains should be promoted by combining neuroimaging, HPA-axis function, inflammatory markers, epigenetic alterations, and process phenotypes such as emotion regulation, attachment patterns, dissociative symptoms, self-injurious behavior, and interpersonal functioning, thereby enhancing the explanatory power of mechanisms linking ACEs and personality disorders. Fourth, future studies should also pay greater attention to protective factors and resilience mechanisms, such as positive childhood experiences, social support, stable caregiving relationships, accessibility of psychotherapy, and the role of trauma-informed interventions in reducing the risk of personality pathology.

From clinical and public health perspectives, the findings of this study suggest that greater attention should be paid to the assessment of adverse childhood experiences among individuals with personality disorders or prominent features of personality pathology. ACEs assessment may help contextualize patients’ developmental backgrounds, particularly in relation to emotion regulation difficulties, interpersonal sensitivity, self-injury risk, and psychiatric comorbidity. It may also provide a basis for trauma-informed assessment, risk formulation, and individualized intervention planning. Future clinical research may further integrate ACEs assessment with evaluations of emotion regulation, attachment patterns, personality functioning, and dimensional personality pathology, thereby promoting a shift in personality disorder-related research from simple diagnostic classification toward mechanism-oriented and individualized models of understanding and intervention. Meanwhile, to facilitate clinical translation, risk prediction models, early identification tools, and intervention evaluation frameworks should be further developed and externally validated, so as to improve the practical value of research findings in clinical screening, prevention strategies, and long-term follow-up management.

## 5 Conclusion

In conclusion, using bibliometric methods, this study provides a comprehensive overview of the global development status, knowledge structure, and evolutionary trends of research on adverse childhood experiences (ACEs) and personality disorders (PDs). The findings indicate that this field has developed rapidly over the past two decades, with increasing international collaboration and a progressively clearer structure of core research themes and knowledge foundations. Research hotspots have gradually expanded from early epidemiological descriptions of the “childhood adversity–personality disorder” exposure–outcome association to deeper issues such as psychiatric comorbidity, intermediate phenotypes, emotion regulation, and neurobiological mechanisms, and have further shown a trend toward translational directions, including risk modeling, diagnostic assessment, and validation studies.

Meanwhile, this field demonstrates a dual knowledge structure in which local citation impact and global citation impact coexist. Some publications constitute the core reference framework within ACEs–PDs research, whereas others have generated broader interdisciplinary influence in fields such as trauma psychiatry, neuroscience, and public health. Overall, this study not only maps the developmental trajectory and research ecology of the field but also provides evidence-based directions for future research. These include conducting longitudinal and cross-cultural studies, strengthening the integration of multimodal mechanistic evidence, focusing on the differential effects of different personality disorder types and ACE subtypes, and promoting clinically oriented studies on risk stratification, early identification, and external validation.

## Data Availability

The original contributions presented in the study are included in the article/**Supplementary Material**. Further inquiries can be directed to the corresponding author.
