# Supplementary material for: Global research landscape, knowledge structure, and emerging trends in adverse childhood experiences and personality disorders: a bibliometric analysis
Source: Front Psychiatry. 2026 Jun 24;17:1842315. doi: 10.3389/fpsyt.2026.1842315 (PMC13342170; doi:10.3389/fpsyt.2026.1842315)
Supplement: Supplementary file 1 [file Table1.docx]

**Table S1. Detailed search strategies for each database**

| **Database** | **Search field** | **Search strategy** | **Limits / notes** |
| --- | --- | --- | --- |
| Web of Science Core Collection | TS | TS=("adverse childhood experience*" OR "childhood adversit*" OR "early life adversit*" OR "early-life adversit*" OR "early life stress*" OR "early-life stress*" OR "childhood trauma*" OR "childhood maltreat*" OR "child maltreat*" OR "child abuse" OR "childhood abuse" OR "child emotional abuse" OR "childhood emotional abuse" OR "child physical abuse" OR "childhood physical abuse" OR "child sexual abuse" OR "childhood sexual abuse" OR "child neglect" OR "childhood neglect" OR "emotional neglect" OR "physical neglect" OR "household dysfunction" OR "family dysfunction" OR "domestic violence" OR "family violence" OR "interparental violence" OR "witnessing domestic violence" OR "exposure to domestic violence" OR "community violence" OR "exposure to community violence" OR bullying OR "peer victimization" OR "childhood victimization" OR "parental separation" OR "parental divorce" OR "parental substance abuse" OR "parental mental illness" OR "parental incarceration" OR "parental loss" OR "parental death" OR "childhood unpredictability" OR "early life unpredictability" OR "unpredictability during childhood" OR "pediatric chronic illness" OR "childhood chronic illness") AND TS=("personality disorder" OR "personality disorders" OR "personality pathology" OR "personality dysfunction" OR "personality disorder traits" OR "borderline personality disorder" OR "emotionally unstable personality disorder" OR "antisocial personality disorder" OR "dissocial personality disorder" OR "narcissistic personality disorder" OR "histrionic personality disorder" OR "avoidant personality disorder" OR "dependent personality disorder" OR "obsessive-compulsive personality disorder" OR "anankastic personality disorder" OR "paranoid personality disorder" OR "schizoid personality disorder" OR "schizotypal personality disorder" OR "cluster A personality disorder" OR "cluster B personality disorder" OR "cluster C personality disorder") | Language: English; document types: Article and Review; publication years: database inception to December 31, 2025. |
| Scopus | TITLE-ABS-KEY | TITLE-ABS-KEY("adverse childhood experience*" OR "childhood adversit*" OR "early life adversit*" OR "early-life adversit*" OR "early life stress*" OR "early-life stress*" OR "childhood trauma*" OR "childhood maltreat*" OR "child maltreat*" OR "child abuse" OR "childhood abuse" OR "child emotional abuse" OR "childhood emotional abuse" OR "child physical abuse" OR "childhood physical abuse" OR "child sexual abuse" OR "childhood sexual abuse" OR "child neglect" OR "childhood neglect" OR "emotional neglect" OR "physical neglect" OR "household dysfunction" OR "family dysfunction" OR "domestic violence" OR "family violence" OR "interparental violence" OR "witnessing domestic violence" OR "exposure to domestic violence" OR "community violence" OR "exposure to community violence" OR bullying OR "peer victimization" OR "childhood victimization" OR "parental separation" OR "parental divorce" OR "parental substance abuse" OR "parental mental illness" OR "parental incarceration" OR "parental loss" OR "parental death" OR "childhood unpredictability" OR "early life unpredictability" OR "unpredictability during childhood" OR "pediatric chronic illness" OR "childhood chronic illness") AND TITLE-ABS-KEY("personality disorder" OR "personality disorders" OR "personality pathology" OR "personality dysfunction" OR "personality disorder traits" OR "borderline personality disorder" OR "emotionally unstable personality disorder" OR "antisocial personality disorder" OR "dissocial personality disorder" OR "narcissistic personality disorder" OR "histrionic personality disorder" OR "avoidant personality disorder" OR "dependent personality disorder" OR "obsessive-compulsive personality disorder" OR "anankastic personality disorder" OR "paranoid personality disorder" OR "schizoid personality disorder" OR "schizotypal personality disorder" OR "cluster A personality disorder" OR "cluster B personality disorder" OR "cluster C personality disorder") AND PUBYEAR < 2026 AND (LIMIT-TO(DOCTYPE, "ar") OR LIMIT-TO(DOCTYPE, "re")) AND LIMIT-TO(LANGUAGE, "English") | Language: English; document types: Article and Review; publication years: database inception to December 31, 2025. |
| PubMed | MeSH and Title/Abstract | ("Adverse Childhood Experiences"[Mesh] OR "Child Abuse"[Mesh] OR "Bullying"[Mesh] OR "Domestic Violence"[Mesh] OR "adverse childhood experiences"[Title/Abstract] OR "adverse childhood experience"[Title/Abstract] OR "childhood adversity"[Title/Abstract] OR "childhood adversities"[Title/Abstract] OR "early life adversity"[Title/Abstract] OR "early-life adversity"[Title/Abstract] OR "early life stress"[Title/Abstract] OR "early-life stress"[Title/Abstract] OR "childhood trauma"[Title/Abstract] OR "childhood maltreatment"[Title/Abstract] OR "child maltreatment"[Title/Abstract] OR "child abuse"[Title/Abstract] OR "childhood abuse"[Title/Abstract] OR "child emotional abuse"[Title/Abstract] OR "childhood emotional abuse"[Title/Abstract] OR "child physical abuse"[Title/Abstract] OR "childhood physical abuse"[Title/Abstract] OR "child sexual abuse"[Title/Abstract] OR "childhood sexual abuse"[Title/Abstract] OR "child neglect"[Title/Abstract] OR "childhood neglect"[Title/Abstract] OR "emotional neglect"[Title/Abstract] OR "physical neglect"[Title/Abstract] OR "household dysfunction"[Title/Abstract] OR "family dysfunction"[Title/Abstract] OR "domestic violence"[Title/Abstract] OR "family violence"[Title/Abstract] OR "interparental violence"[Title/Abstract] OR "witnessing domestic violence"[Title/Abstract] OR "exposure to domestic violence"[Title/Abstract] OR "community violence"[Title/Abstract] OR "exposure to community violence"[Title/Abstract] OR bullying[Title/Abstract] OR "peer victimization"[Title/Abstract] OR "childhood victimization"[Title/Abstract] OR "parental separation"[Title/Abstract] OR "parental divorce"[Title/Abstract] OR "parental substance abuse"[Title/Abstract] OR "parental mental illness"[Title/Abstract] OR "parental incarceration"[Title/Abstract] OR "parental loss"[Title/Abstract] OR "parental death"[Title/Abstract] OR "childhood unpredictability"[Title/Abstract] OR "early life unpredictability"[Title/Abstract] OR "unpredictability during childhood"[Title/Abstract] OR "pediatric chronic illness"[Title/Abstract] OR "childhood chronic illness"[Title/Abstract]) AND ("Personality Disorders"[Mesh] OR "personality disorder"[Title/Abstract] OR "personality disorders"[Title/Abstract] OR "personality pathology"[Title/Abstract] OR "personality dysfunction"[Title/Abstract] OR "personality disorder traits"[Title/Abstract] OR "borderline personality disorder"[Title/Abstract] OR "emotionally unstable personality disorder"[Title/Abstract] OR "antisocial personality disorder"[Title/Abstract] OR "dissocial personality disorder"[Title/Abstract] OR "narcissistic personality disorder"[Title/Abstract] OR "histrionic personality disorder"[Title/Abstract] OR "avoidant personality disorder"[Title/Abstract] OR "dependent personality disorder"[Title/Abstract] OR "obsessive-compulsive personality disorder"[Title/Abstract] OR "anankastic personality disorder"[Title/Abstract] OR "paranoid personality disorder"[Title/Abstract] OR "schizoid personality disorder"[Title/Abstract] OR "schizotypal personality disorder"[Title/Abstract] OR "cluster A personality disorder"[Title/Abstract] OR "cluster B personality disorder"[Title/Abstract] OR "cluster C personality disorder"[Title/Abstract]) | Language: English; publication years: database inception to December 31, 2025. Publication types were harmonized during post-retrieval screening. |
